# Reversible Stress‐memory Phosphorescent Carbon Nanodots via Supramolecular Confinement Engineering for Aerospace Monitoring

**DOI:** 10.1002/advs.202521219

**Published:** 2025-12-12

**Authors:** Yachuan Liang, Haochun Shao, Kaikai Liu, Qing Cao, Liying Jiang, Chongxin Shan, Leman Kuang, Hui Jing

**Affiliations:** ^1^ School of Electronics and Information Zhengzhou University of Light Industry Zhengzhou 450002 China; ^2^ Academy for Quantum Science and Technology Zhengzhou University of Light Industry Zhengzhou 450002 China; ^3^ Henan Key Laboratory of Information Functional Materials and Sensing Technology Zhengzhou University of Light Industry Zhengzhou 450002 China; ^4^ Henan Key Laboratory of Diamond Optoelectronic Material and Devices School of Physics and Laboratory of Zhongyuan Light Zhengzhou University Zhengzhou 450001 China; ^5^ Key Laboratory of Low‐Dimensional Quantum Structures and Quantum Control of Ministry of Education Department of Physics and Synergetic Innovation Center for Quantum Effects and Applications Hunan Normal University Changsha 410081 China

**Keywords:** aerospace monitoring, carbon nanodots, mechano‐responsive, phosphorscence, supramolecular confinement engineering

## Abstract

The development of mechano‐responsive room‐temperature phosphorescent (RTP) materials with reversibility and durable memory stress‐recording capability remains a critical challenge, particularly under extreme operational conditions where covalent bond‐dependent systems often suffer from irreversible degradation. Herein, a hydrogen‐bond‐induced dynamic supramolecular confinement framework is constructed to achieve cyclodextrin‐trapped carbon nanodots (CNDs) with reversible and memorable mechano‐responsive RTP. Mechanical stress disrupts the metastable hydrogen‐bond network and weakens phosphorescence via enhanced non‐radiative decay of triplet excitons. Remarkably, the system exhibits a recovery of RTP intensity through ultrasonic reconstruction of the rigid cyclodextrin matrix. When deployed in aerospace structural health monitoring, the CND‐embedded film visualizes stress distribution in wings under sudden stress events through RTP weakening. This work establishes a non‐destructive monitoring paradigm for an extreme aerospace environment.

## Graphical abstract

A kind of new CNDs‐based materials with reversible mechano‐responsive phosphorescence emission through incorporating CNDs into the supramolecular rigid network is leveraged for aerospace applications, where the CND‐embedded film demonstrated memorable visualization of stress distribution in wings under sudden mechanical events (e.g., micro‐impacts).

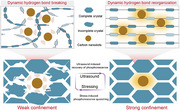

Mechano‐responsive luminescent materials, which can transduce mechanical stimuli into optical signals, have revolutionized stress‐sensing technologies. These materials offer a method for monitoring structural integrity remotely, with applications spanning aerospace composites, artificial skins, and anti‐counterfeiting systems.^[^ ^1^ ^]^ Their ability to visually indicate mechanical stress has opened up new possibilities in aerospace, enabling more efficient and precise tracking of potential material failure points and deformation. However, their transient photon emission and irreversible energy dissipation prevent the recording of mechanical events that occur at unpredictable or infrequent intervals. Such events include high‐impact collisions in automotive safety systems or sudden stress on structural components in aerospace materials. The inability to store these transient events limits the application of mechano‐responsive luminescent materials in scenarios where historical data are essential for ensuring reliability and safety. Many conventional materials, such as ZnS: Mn‐based phosphors,^[^ ^2^, ^3^, ^4^ ^]^ suffer from luminescence instantaneity under mechanical stress, which is often attributable to the rapid radiative relaxation of excited electrons caused by lattice vibrations. The Kim group has achieved luminescent changes under different mechanical forces. However, we now note that their luminescent signals are often unstable and tend to relax or decay rapidly after the force is removed, which severely limits their practical application in long‐term data storage.^[^ ^2^ ^]^ Moreover, the rigid crystalline nature of inorganic phosphors significantly limits their mechanical processability, making them incompatible with the flexible architectures required for wearable application scenarios. Suhr group developed excellent mechano‐responsive materials that, while their systems are highly responsive, the written luminescent information is typically permanent and cannot be erased or rewritten, confining their practicality.^[^ ^4^ ^]^ In addition, organic materials, while offering improved flexibility and processability, often display poor environmental stability, being susceptible to photobleaching or thermal degradation.^[^ ^5^ ^]^ Consequently, there remains an urgent need to engineer new classes of mechano‐responsive luminophores that combine optical reversibility and storage function.^[^ ^6^ ^]^

Carbon nanodots (CNDs), as emerging 0D carbon‐based luminescent materials, have garnered significant attention due to their tunable emission, low toxicity, and facile surface functionalization, enabling applications in bioimaging, optoelectronics, and sensing.^[^ ^7^, ^8^, ^9^ ^]^ Recently, mechano‐responsive fluorescent materials have been developed by incorporating emitters into polymer matrices or supramolecular networks, where pressure‐induced aggregation or bond‐breaking modulates fluorescence intensity.^[^ ^10^, ^11^ ^]^ However, such systems face critical limitations: i) ‌Irreversible structural damage‌ under cyclic loading results in rapid performance degradation; ii) ‌Short‐lived emission‌ restricts their utility in scenarios requiring persistent optical signals. Compared to fluorescence, room‐temperature phosphorescence (RTP) of CNDs offers distinct advantages for mechanical sensing: i) ‌Long‐lived emission‌ (milliseconds to seconds) allows time‐gated detection to eliminate autofluorescence interference; ii) ‌Triplet exciton sensitivity‌ to microenvironments enables higher pressure resolution; iii) ‌Stable optical memory effect‌ permits post‐event mechanical history retrieval. However, achieving mechano‐responsive RTP in CNDs remains challenging.^[^ ^12^, ^13^, ^14^, ^15^ ^]^ While mechanical deformation can modulate phosphorescence intensity, most systems fail to retain the original optical memory of stress history, which arises from permanent damage to exciton‐protecting sites during compression. Moreover, external stimuli (e.g., stress) that perturb the rigid matrix often irreversibly destroy triplet exciton protection sites. Therefore, the development of mechano‐responsive RTP CNDs with ‌a stable optical memory effect‌ that permits post‐event mechanical history retrieval is of significant importance for their potential applications.

Achieving mechano‐responsive phosphorescence with reversible and memorable features relies on the interaction between exciton stabilization and dynamic structural adaptability. In this respect, supramolecular confinement provides a strategy to balance these competing requirements.^[^ ^16^, ^17^, ^18^, ^19^, ^20^, ^21^, ^22^, ^23^ ^]^ Confined interaction within the matrix suppresses non‐radiative pathways, stabilizing the triplet excitons of CNDs and enabling ultralong RTP.^[^ ^1^, ^9^, ^24^, ^25^, ^26^, ^27^, ^28^, ^29^, ^30^, ^31^, ^32^, ^33^, ^34^ ^]^ Crucially, a stress‐sensitive hydrogen‐bond network formed between CNDs and the supramolecular host allows mechanical forces to modulate optical properties.^[^ ^5^, ^22^, ^35^ ^]^ Under applied stress, reversible distortion of the hydrogen bonds leads to a substantial increase in non‐radiative decay rates, resulting in proportional quenching of phosphorescence. The mechanically perturbed state is maintained through metastable interactions, effectively memorizing the mechanical history in the optical signal. Moreover, recovery is achieved by reconstruction of the hydrogen bonds through ultrasonic stimulation, which resets the metastable interactions without compromising the overall confinement.

Herein, we report mechano‐responsive RTP CNDs achieved by constructing a dynamically cyclodextrin confinement framework through hydrogen‐bond‐induced supramolecular assembly. The CNDs exhibit weakened phosphorescence under mechanical stress due to distortion‐induced disruption of the hydrogen‐bond network, which enhances non‐radiative decay of the triplet exciton. Remarkably, subsequent ultrasonic treatment reconstructs the confinement framework, enhancing the RTP intensity by re‐establishing the spatial confinement of CNDs within the rigid cyclodextrin matrix.^[^ ^20^, ^36^, ^37^, ^38^, ^39^, ^40^, ^41^, ^42^ ^]^ This reversible “stress‐recording” behavior originates from the metastable yet robust hydrogen‐bond interactions that preserve the triplet exciton without covalent bond cleavage.^[^ ^2^, ^43^, ^44^, ^45^ ^]^ When integrated into aerospace instantaneous stress monitoring, the CND‐embedded film demonstrates instantaneous phosphorescence weakening under sudden stress events (e.g., micro‐impact), enabling visualization of stress distribution in the wing, which establishes a paradigm for non‐destructive structural health monitoring in extreme aerospace environments.

Triplet excitons are sensitive to their surrounding environment, with the rigidity of the confined microenvironment playing a pivotal role in governing their radiative and non‐radiative decay. A highly rigid environment can effectively suppress non‐radiative deactivation, thereby prolonging the excited‐state lifetime and enhancing phosphorescent emission.^[^ ^36^ ^]^ However, achieving precise control over microenvironmental rigidity in solid‐state materials remains a significant challenge. In this study, we present a strategy to dynamically regulate the microenvironment of triplet excitons by embedding carbon nanodots (CNDs) in cyclodextrin crystalline frameworks (Figure S1, Supporting Information). Cyclodextrin‐trapped CNDs exhibit ultralong room‐temperature phosphorescence (RTP) by leveraging a hydrogen‐bond‐stabilized rigid microenvironment (Figures S2–S4, Supporting Information). The hydrophobic cavities of cyclodextrin molecules spatially isolate CNDs, effectively restricting their molecular motion and shielding the triplet states from environmental quenchers. This tight confinement suppresses non‐radiative decay, thus stabilizing the triplet excited states and enabling an efficient RTP. Moreover, the unique framework may endow the system with mechano‐responsive properties. When applying mechanical stress, the hydrogen‐bond network anchoring the CNDs undergoes reversible distortion or breaking (**Figure** **1**a left). This stress‐induced structural perturbation weakens the rigidity of the microenvironment, increasing the rotational and vibrational freedom of CNDs and significantly enhancing the non‐radiative decay rate. As a result, the weakened phosphorescence intensity is observed (Figure 1b left). Importantly, the mechanically induced state is preserved via metastable hydrogen‐bond interactions, although sufficiently labile to allow dynamic deformation, are robust enough to maintain the triplet excitons without involving covalent bond cleavage. This metastable state enables the mechanical stress to be memorized optically, encoding the mechanical history into the phosphorescent signal. Notably, the mechanically perturbed state can be erased through ultrasonic stimulation, which provides energy to enhance the metastable hydrogen‐bond interactions, while reconstructing the structural integrity of the confinement framework (Figure 1a right). Following ultrasonic stimulation, the rigidity of the environment is restored, the non‐radiative decay rate decreases, and the phosphorescence intensity is enhanced (Figure 1b right), highlighting the stability and re‐configurability of the hydrogen‐bonded framework.

**Figure 1:**
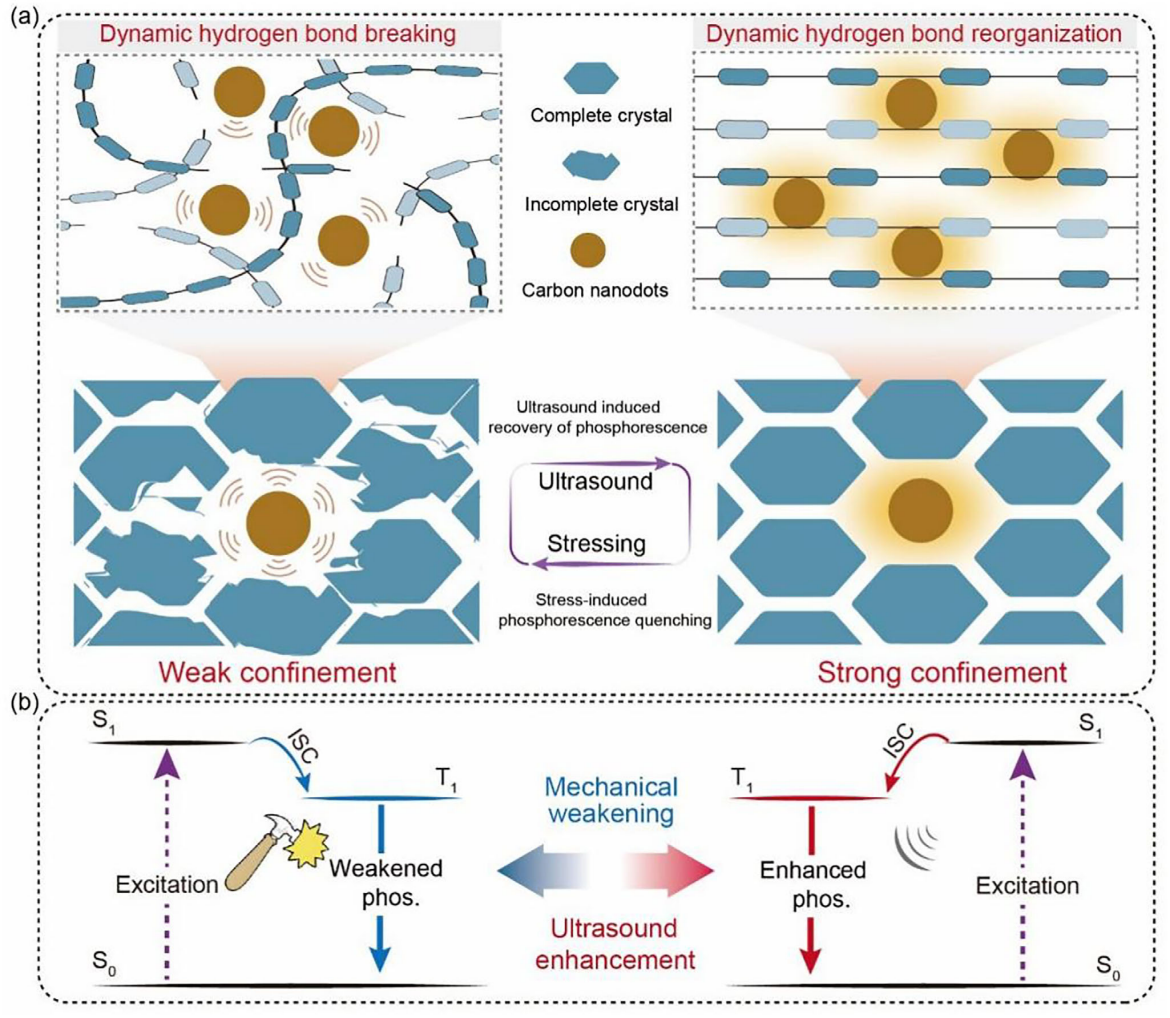
Phenomenon and mechanism of the cyclodextrin‐trapped CNDs. a,b) Schematic illustration of the mechano‐responsive phosphorescence mechanism (a) and corresponding energy level transition diagram (b).

To validate our hypothesis, we selected phosphorescent carbon nanodots (CNDs) as the luminescent emitters and investigated their mechano‐responsive behavior with cyclodextrin under mechanical stress. First, bare CNDs were synthesized and characterized. By studying the RTP performance of the CNDs and cyclodextrin assembly systems with different ratios, the optimal parameters for the cyclodextrin‐trapped CNDs are determined: 1.4 mL CNDs aqueous solution and 1 g cyclodextrin in deionized water (DI) to form an aqueous solution (Figure S5, Supporting Information). Transmission electron microscopy (TEM, Figure S6, Supporting Information) revealed that the average diameter of the bare CNDs was ≈3 nm. The optical properties of the bare CNDs were recorded using UV–vis absorption, photoluminescence (PL), phosphorescence spectroscopy, and excitation‐emission mapping. As shown in Figure S7 (Supporting Information), the fluorescence spectrum exhibited an emission peak at 445 nm, while no detectable phosphorescence signal was observed (Figure S8, Supporting Information). The inset displays the corresponding fluorescence and phosphorescence images. The UV–vis absorption spectrum (Figure S9a, Supporting Information) revealed two distinct absorption peaks at ≈280 and 350 nm, which can be attributed to π–π^*^ and n–π^*^ electronic transitions, respectively. Given the close correspondence between the excitation (Figure S10, Supporting Information) and absorption spectra, it is likely that the fluorescence emission arises primarily from the n–π^*^ transition. Additionally, the fluorescence lifetime of the CNDs was measured at 445 nm and is shown in Figure S9b (Supporting Information). The fitted fluorescence lifetime was determined to be 4.2 ns. The excitation‐emission contour plot (Figure S11, Supporting Information) further indicates that the CNDs exhibit excitation wavelengths ranging from 350 to 400 nm, with an optimal excitation wavelength centered at 375 nm. To investigate the mechano‐responsive behavior of RTP in cyclodextrin‐trapped CNDs, a custom‐designed mechanical compression device was constructed Figure S12 (Supporting Information). This apparatus enables the application of quantifiable external pressure to solid‐state samples. And the relationship between mechanical stress and the phosphorescence properties of cyclodextrin‐trapped CNDs was explored. **Figure** **2**a presents a series of RTP spectra of cyclodextrin‐trapped CNDs recorded under varying levels of mechanical stress. As the stress increases, the RTP intensity centered at 515 nm exhibits a monotonic decline. The stress‐dependent modulation of phosphorescence intensity can be quantitatively described by the non‐radiative decay rate constant (k_nr_):

(1) $$k_{\mathrm{nr}}=A_{\exp}\left(-E_{a}/k_{B}T\right)+B\sigma$$

**Figure 2:**
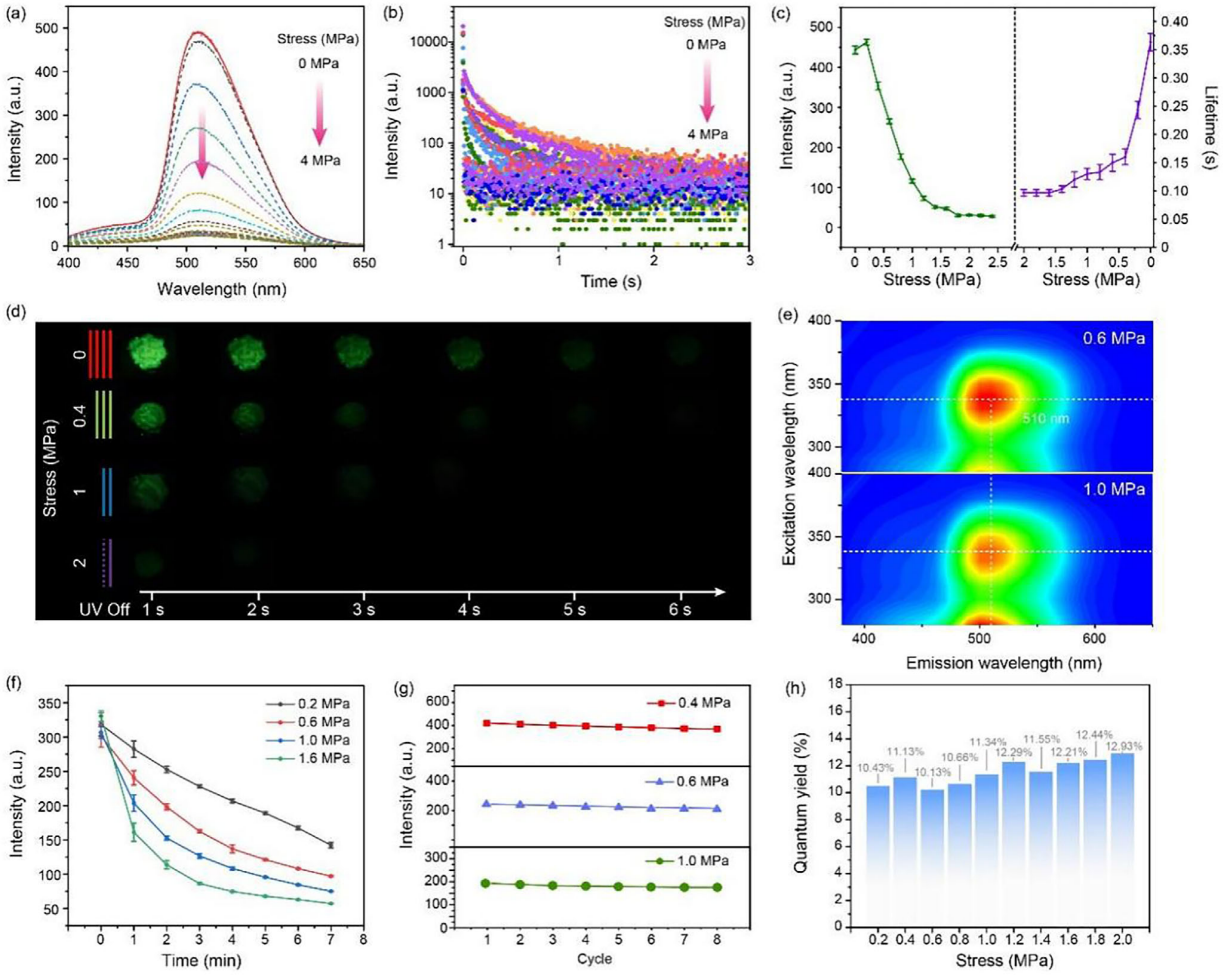
Stress‐dependent modulation and memory effect of RTP in cyclodextrin‐trapped CNDs. a) Phosphorescent spectra of the cyclodextrin‐trapped CNDs under increasing mechanical stress. b) Phosphorescence lifetime decay curves under varying stress. c) Quantitative summary of RTP intensity and lifetime as functions of applied stress. d) Phosphorescence photographs of the cyclodextrin‐trapped CNDs after different stresses. e) The excitation‐phosphorescence mapping of the cyclodextrin‐trapped CNDs after different stresses. f) The relationship between phosphorescence intensity and exposure duration under different stresses. g) Repetitive excitation cycles of loading samples. h) The quantum yield of cyclodextrin‐trapped CNDs after different stresses.

This formulation confirms that mechanical stress (σ) directly enhances k_nr_ by reducing the effective energy barrier (E_a_) through hydrogen‐bond network distortion. The substantial quenching of phosphorescence (Figure 2a) indicates a mechanical stress‐induced perturbation of the triplet state dynamics. Specifically, pressure enhances non‐radiative decay pathways for triplet excitons, such as increased vibrational relaxation and interactions with surrounding matrix molecules, which compete with phosphorescence emission and suppress the phosphorescence intensity. Mechanical stress induces distortion or breaking of the hydrogen‐bond network between cyclodextrin and CNDs, which weakens the rigidity of the supramolecular matrix, increasing vibrational/rotational freedom of CNDs and promoting vibrational/rotational motions that were previously restricted. This newly molecular mobility creates non‐radiative decay pathways. The radiative energy is dissipated as heat through these enhanced vibrational modes (internal conversion), which may lead to the observed quenching of the luminescence intensity. Importantly, the spectral profiles remain consistent in shape across all pressure levels, indicating that the emission originates from the same emissive triplet state and that no new radiative transitions are introduced or alter the electronic structure of the CNDs under mechanical stress. Instead, the primary effect of increasing pressure is a reduction in peak intensity, reflecting the relationship between pressure and phosphorescence intensity. A detailed assessment of the stress‐dependent lifetime changes is presented in Figure 2b. At ambient conditions (0 MPa), the sample exhibits a long‐lived phosphorescence lifetime of ≈0.36 s. Upon increasing pressure to 4 MPa, the lifetime progressively shortens to 96 ms, with the most dramatic reductions occurring between 0–2 MPa. This phenomenon is consistent with the pressure‐induced perturbation of the hydrogen‐bonded network surrounding the CNDs. Figure 2c summarizes the decrease in both RTP intensity and lifetime as functions of applied stress. This simultaneous reduction in intensity and lifetime reveals a stress‐dependent correlation between the rigidity of the CNDs microenvironment and the efficiency of triplet‐state emission. This quenching behavior is attributed to the disruption of the rigid, hydrogen‐bond‐stabilized microenvironment formed by the cyclodextrin framework. As pressure is applied, the hydrogen bonding network undergoes structural deformation, reducing its ability to restrict molecular motion, thereby facilitating vibrational relaxation and diminishing the phosphorescence emission. As shown in Figure 2d, the sample displays bright green phosphorescence persisting several seconds after UV excitation ceases at 0 MPa. As stress increases, the phosphorescent emission gradually fades, consistent with the spectroscopic data. Notably, both spectra (After different pressures) exhibit similar peak positions (Figure 2e), with consistent excitation and emission maxima, indicating that the electronic structure and radiative transitions of the CNDs remain unchanged under mechanical stress. However, a decrease in overall intensity is observed at the higher pressure. The decrease in phosphorescence emission, without spectral shift, confirms that the applied pressure predominantly affects the rigidity of the microenvironment around the CNDs rather than the inherent photophysical properties of the CNDs. To investigate the effects of pressure magnitude and duration, phosphorescence intensity was monitored under multiple exposure conditions (Figure 2f). A consistent decline in phosphorescence intensity is observed with increasing stress, which becomes more pronounced with longer exposure durations. This behavior reflects the gradual degradation of the rigid hydrogen‐bonded framework under stress, resulting in enhanced non‐radiative relaxation. Interestingly, as shown in unloaded spectra, the phosphorescence does not recover spontaneously upon removal of the external pressure, even after 30 min of ambient relaxation, demonstrating a memory effect (Figure S13, Supporting Information). The emission profile remains nearly identical to that measured after stress, confirming the presence of a mechanically induced metastable state (Figure S14, Supporting Information). This memory effect is attributed to the rearrangement of hydrogen bonds within the cyclodextrin framework, which cannot easily revert to their original configuration without external input. To validate the stability of this mechano‐optical memory, a cyclic experiment was conducted using independent samples pre‐stressed at 0.4, 0.6, and 1 MPa. Each sample was subjected to multiple excitation cycles (Figure 2g). The phosphorescence intensity remained constant within each group across cycles, demonstrating that the RTP signal reliably encodes the mechanical history. This memorability originates from a metastable hydrogen‐bond network, which encodes the history of mechanical stress into the optical response. The ability to retain optical states over time without structural damage underscores the potential of this system for optical memory and stress‐logging applications. In contrast to the stress‐dependent trends in intensity and lifetime, the quantum yield (QY) remains essentially unchanged across the entire stress range (Figure 2h). Measured absolute QY values fluctuate within a narrow range, confirming that the observed changes in emission are not due to degradation of the CNDs. Instead, the modulation of phosphorescence is governed exclusively by changes in the rigidity of the matrix around the CNDs. The preservation of intrinsic photophysical efficiency supports that the cyclodextrin framework, not the luminescent center, is the primary factor mediating mechano‐responsiveness.

As shown in **Figure** **3**a, the fluorescence spectra of cyclodextrin‐trapped CNDs remain unchanged before and after mechanical stressing, indicating that the electronic structure of the CNDs are not perturbed by applied pressure. However, the corresponding phosphorescence is quenched, revealing a pronounced sensitivity of triplet‐state emission to mechanical deformation of the surrounding matrix. This observation underscores that while the luminescent center itself remains intact, the rigidity of the local microenvironment is compromised, leading to increased non‐radiative decay pathways. Remarkably, the quenched phosphorescence can be restored via ultrasonic treatment, as illustrated in Figure 3a. Upon subjecting the stress‐treated samples to ultrasond, a gradual recovery of phosphorescence intensity is observed (Figure 3b), with enhancement positively correlated with treatment duration (0–40 min), which suggests that ultrasound facilitates the reconstruction of the hydrogen‐bond‐stabilized cyclodextrin framework, thereby re‐establishing the rigid confinement necessary for RTP of CNDs. The increase in phosphorescence intensity demonstrates that the mechanically perturbed state is reversible under external ultrasonic stimuli. Moreover, the phosphorescence lifetime also recovers during ultrasonic treatment, as shown in Figure 3c. The lifetime increases from 0.11 to 0.17 s. The recovery of phosphorescence and lifetime upon ultrasonic treatment indicates that the rigid microenvironment required to suppress non‐radiative relaxation is successfully reconstructed (Figure 3d). For comparison, similar experiments were conducted using SiO_2_‐encapsulated CNDs. After mechanical stressing of CNDs@SiO_2_ powder (Figure S15, Supporting Information), a partial quenching of phosphorescence was observed, but the degree of suppression was less than in the cyclodextrin matrix. Subsequent ultrasonic treatment cannot recovery of RTP intensity. Unlike the permanent damage typically observed in conventional rigid matrix, the cyclodextrin enables reversible structural reconfiguration, driven by non‐covalent interactions that are both mechano‐sensitive and ultrasound‐responsive. To further validate this mechano‐responsive reversibility, Figure 3e presents a cyclic experiment in which samples are subjected to stress and ultrasonic recovery over five cycles. The phosphorescence intensity remains consistent throughout the cycles, with no observable degradation. This robust cyclic stability indicates the reversible and repeatable nature of the confined interaction‐driven phosphorescence and demonstrates the potential for applications in reversible optical memory and stress sensing. The reported works of mechano‐responsive luminescent materials are collected, and the corresponding luminescent wavelengths, memorability, and restorability are summarized in **Table** **1**. To investigate the mechanism of ultrasound‐induced recovery, the effect of ultrasonic power on the recovery kinetics was studied. As shown in Figure 3f, higher power levels lead to faster and more pronounced enhancement in phosphorescence intensity for the same exposure time, which suggests that stronger ultrasonic energy accelerates the reassembly of the rigid hydrogen‐bonded framework necessary for phosphorescence emission. In addition, the transmission electron microscopy (TEM) image (Figure 3g) reveals the highly completed crystalline morphology of the cyclodextrin‐trapped CNDs, which confirms successful supramolecular crystallization driven by hydrogen bond between cyclodextrin hosts and CNDs, facilitating efficient phosphorescence emission. Upon mechanical stressing, the crystalline structure is significantly disrupted. The loss of lattice periodicity and appearance of amorphous regions indicate fracture of hydrogen‐bonded networks, leading to diminished phosphorescence due to the breakdown of confinement‐induced stabilization. After ultrasonic treatment, the crystalline structure largely reverts to its original crystalline state, which confirms the reversible nature of the hydrogen‐bonded assembly and correlates with the regeneration of enhanced phosphorescence, underscoring the role of dynamic hydrogen‐bonded supramolecular confinement in the memorizable mechano‐responsive phosphorescence. The above results indicate that cyclodextrin‐trapped CNDs form a reversible phosphorescent system that not only responds to mechanical stress but also exhibits ultrasound‐triggered recovery, enabled by the metastable nature of its hydrogen‐bonding network.

**Figure 3:**
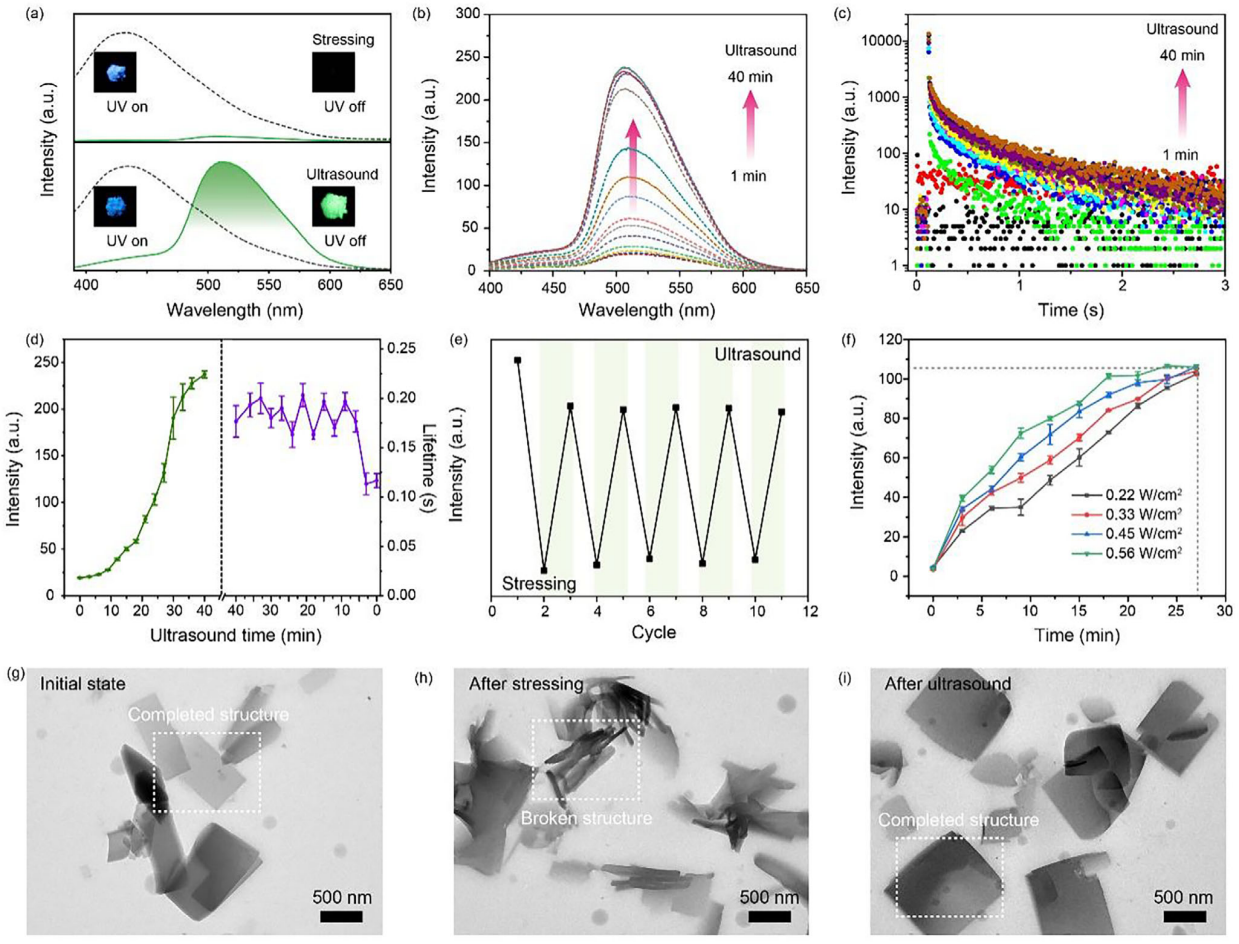
Ultrasound‐induced recovery of RTP in cyclodextrin‐trapped CNDs. a) The fluorescence and phosphorescence spectra of cyclodextrin‐trapped CNDs after stressing and ultrasound treatment. b) Time‐dependent recovery of RTP intensity under ultrasonic treatment. c) Phosphorescence lifetime decay curves under varying ultrasonic treatment. d) Quantitative summary of RTP intensity and lifetime as functions of varying ultrasonic treatment. e) RTP intensity of cyclodextrin‐trapped CNDs over five mechanical stress‐ultrasound recovery cycles. f) Dependence of recovery efficiency on ultrasonic power. g) TEM image of the cyclodextrin‐trapped CNDs. h) TEM image of the cyclodextrin‐trapped CNDs after applying mechanical stressing. i) TEM image of mechano‐treated cyclodextrin‐trapped CNDs after ultrasonic treatment.

**Table 1:** Reported Work of Mechano‐responsive Luminescent Materials.

Reference	Materials	Luminescence	Wavelength	Memorability	Restorability
[46]	PMA_162_	Fluorescence	390 nm	√	×
[47]	PCL‐An‐PU‐TAD	Fluorescence	435 nm	√	×
[48]	TPE‐4N	Fluorescence	520 nm	√	×
[49]	TPE‐CN	Fluorescence	468 nm	×	×
[50]	TPE‐4N@PDMS	Fluorescence	525 nm	×	√
[51]	o‐Nacpb	Phosphorescence	497 nm	×	√
[12]	TpNP_0.5_@PU_51_	Phosphorescence	504 nm	×	√
This work	CNDs	Phosphorescence	515 nm	√	√

In order to reveal the mechanism of the mechano‐responsive phosphorescence of cyclodextrin‐trapped CNDs, a series of structural and spectroscopic analyses was performed. As shown in **Figure** **4**a and X‐ray powder diffraction (XRD) reveals that the crystallinity of the cyclodextrin‐trapped CNDs decreases progressively with increasing mechanical stress. Specifically, the diffraction peaks weaken as stress increases, suggesting disruption of the crystalline lattice within the cyclodextrin framework. This loss of crystallinity is likely due to the distortion of the hydrogen‐bonded network that confines the CNDs. The reduction in crystallinity diminishes the rigidity of the local environment, thereby promoting non‐radiative decay. Interestingly, when subjecting to ultrasonic treatment, the diffraction peaks gradually enhance with increasing exposure time. This recovery in crystallinity implies that ultrasond promotes the restruction of the hydrogen bonding network, effectively restructing the rigid microenvironment essential for RTP. This structural reversibility provides compelling evidence for the recoverable mechano‐responsive nature of the cyclodextrin‐trapped CNDs. In addition, a pressure‐dependent increase in Electron paramagnetic resonance (EPR) signal intensity is observed (Figure 4b), which indicates that the structural perturbation of the cyclodextrin matrix under stress promotes interactions between CNDs and the surrounding environment, thereby facilitating the energy transfer process from triplet excited state to the singlet oxygen state, ultimately leading to phosphorescence quenching. The enhanced non‐radiative decay under stress follows Fermi's Golden Rule:

**Figure 4:**
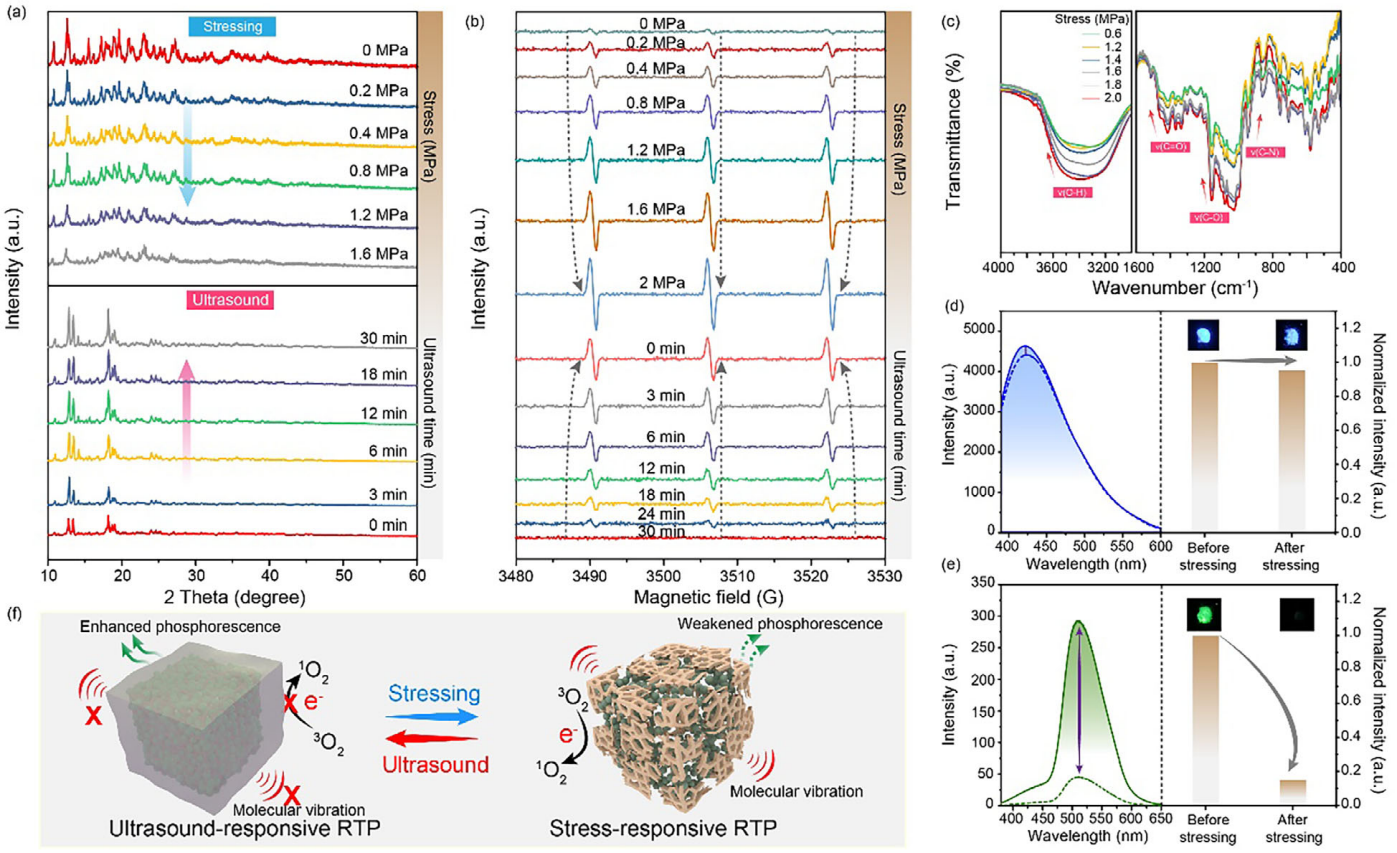
Mechanism investigation of mechano‐responsive phosphorescence in cyclodextrin‐trapped CNDs. a) XRD patterns of cyclodextrin‐trapped CNDs under different mechanical stresses and after ultrasonic recovery. b) EPR spectra of cyclodextrin‐trapped CNDs under different mechanical stresses and after ultrasonic recovery. c) FTIR spectra of cyclodextrin‐trapped CNDs under different mechanical stresses. d) Fluorescence spectra of cyclodextrin‐trapped CNDs before and after mechanical stress. e) Phosphorescence spectra of cyclodextrin‐trapped CNDs before and after mechanical stress. f) Schematic illustration of the mechano‐responsive RTP mechanism.

(2) $$k_{\mathrm{nr}}\propto |<\psi_{f}|H^{\prime }|\psi_{i}>{|}^{2}\rho$$

where *H*′ represents the stress‐perturbed Hamiltonian, ψ*_i_* and ψ*_f_* are initial/final exciton states, and *ρ* is the density of states. The disruption of the rigid structure facilitates the diffusion of oxygen molecules and makes them closer to the excited‐state CNDs. Specifically, the excited triplet state of CNDs can efficiently transfer its energy to the triplet ground state of molecular oxygen (^3^O_2_), generating singlet oxygen (^1^O_2_). The increased EPR signal confirms *H*′ mediates triplet‐to‐singlet oxygen energy transfer. Notably, after ultrasonic treatment, the EPR signal intensity gradually decreases with increasing treatment duration, likely by reconstructing the rigid environment to suppress interactions between CNDs and molecular oxygen, thereby decrease the yield of singlet oxygen. Fourier transform infrared (FTIR) spectroscopy was used to analyze changes in chemical interactions within the cyclodextrin‐trapped CNDs. As shown in Figure 4c, stress leads to a noticeable reduction in the C─O stretching vibration near 1180 cm^−1^, suggesting weakening of hydrogen bonding interactions between CNDs and cyclodextrin. In addition, the broad O─H stretching band centered ≈3300 cm^−1^ also decreases with increasing stress, indicating a loss of hydrogen‐bonding interactions. These results imply that mechanical stress disrupts the hydrogen‐bond network, reducing the rigid of the cyclodextrin matrix. To further investigate the effect of stress on photophysical behavior, fluorescence and phosphorescence spectra were recorded before and after mechanical stress. As shown in Figure 4d, the fluorescence spectra remain nearly unchanged after stress, confirming that the electronic structure of the CNDs are not affected. However, a distinct reduction in phosphorescence is observed (Figure 4e), consistent with the loss of a rigid confinement necessary to stabilize triplet excitons. No shift in emission wavelength supports the hypothesis that non‐radiative decay pathways are enhanced due to microstructural deformation rather than changes in the electronic structure of the CNDs.

Overall, we propose a reasonable mechanism for the mechano‐responsive phosphorescence of cyclodextrin‐trapped CNDs. As shown in Figure 4f, the mechano‐responsive phosphorescence of cyclodextrin‐trapped CNDs arises from stress‐induced structural perturbations in the rigid matrix. Under mechanical stress, the hydrogen‐bonded network between cyclodextrin and CNDs undergoes disruption, which weakens the rigid microenvironment critical for suppressing non‐radiative decay of triplet excitons, thereby enhancing vibration/rotation of CNDs and promoting energy transfer from the triplet excited state to molecular oxygen. Consequently, the phosphorescence intensity decreases under stressing due to competing non‐radiative pathways. Notably, the mechano‐responsive phosphorescence is reversible. Upon ultrasonic treatment, vibrational energy facilitates the lattice reassembly. The restored rigidity hinders interactions between triplet excitons and oxygen while suppressing vibration/rotation of CNDs. The stress‐memory effect originates from metastable hydrogen‐bond configurations described by a double‐well potential model:

(3) $$V\left(x\right)=V_{0}\left[{\left(x^{2}/a^{2}-1\right)}^{2}+\left(\sigma /\sigma_{c}\right)x/a\right]$$

where x is the reaction coordinate, σ_c_ is the critical stress, and a is the characteristic length. Mechanical loading tilts the potential (σ > 0), trapping the system in a local minimum that persists post‐unloading. The persistence of the metastable state is attributed to the irreversible (or kinetically hindered) structural reorganization of the cyclodextrin‐trapped CNDs upon mechanical stress. This process, which may include phenomena such as amorphization or disruption of crystalline packing, creates a new local energy minimum. Crucially, the thermal energy (K_B_T) at room temperature is insufficient to provide the activation energy required for the system to spontaneously revert to its original state. Therefore, once the mechanical stress is removed, the system remains maintained in this metastable configuration and retains the structural memory of the pressure‐induced interactions, resulting in the memorable mechano‐responsive luminescence. Ultrasonic stimulation provides the energy to overcome the barrier ΔV, restoring the original configuration. Crucially, the mechano‐responsive behavior is governed by dynamic structural rearrangements in the cyclodextrin matrix, which regulate the rigidity‐dependent triplet exciton dynamics. The ultrasonic energy provides the requisite activation through the mechanical perturbation mechanism. When ultrasonic waves propagate through the material, they generate localized mechanical vibrations and stress waves. This mechanical energy generates intense local energy fields. The mechanical energy from ultrasonic waves is converted into vibrational energy, which increases the thermal energy of the system. This energy input is utilized by the system to overcome the activation barrier (ΔV). Specifically, the mechanical impact of these shockwaves provides a directed force that disrupts the metastable molecular arrangements of cyclodextrin, effectively perturbing the system and pushing it over the energy barrier, which allows the cyclodextrin molecules to relax back to the thermodynamically stable rigid state, restoring the original confinement properties. This confined interaction‐driven mechanism enables a reversible, stress‐memorizable phosphorescence response. In addition, the stability of CNDs is a key factor for the practical applications. To evaluate the photostability of cyclodextrin‐trapped CNDs, the sample was continuously irradiated by a 350 nm light source for up to 80 min (Figure S16a, Supporting Information), and the phosphorescence intensity maintained more than 90% of its initial value, demonstrating good resistance to photobleaching. Furthermore, temporal stability was assessed by storing the cyclodextrin‐trapped CNDs at room temperature for 10 days, and the phosphorescence intensity was recorded at regular intervals. The results showed minimal change in the emission intensity over time, indicating excellent temporal stability (Figure S16b, Supporting Information).

To demonstrate the potential of the as‐prepared material in stress‐recording applications, cyclodextrin‐trapped CNDs were embedded in a polyvinyl alcohol (PVA) matrix to fabricate a flexible luminescent film (CND‐PVA film), as illustrated in **Figure** **5**a. This film serves as a mechano‐responsive optical platform capable of recording mechanical stimuli via changes in phosphorescent intensity. Figure S17 (Supporting Information) displays the photoluminescent spectrum of the CND‐PVA film, showing an emission peak that is close to that of the pristine CNDs (i.e., without PVA encapsulation). This spectral consistency indicates that incorporation into the PVA matrix does not alter the intrinsic luminescent properties of the CNDs. The phosphorescence decay profile, shown in Figure S18 (Supporting Information), reveals a long‐lived emission with a lifetime of 0.81 s, confirming the ability of the PVA matrix to preserve the confined environment necessary for efficient phosphorescence. The durability of the film under mechanical deformation was examined by subjecting it to 500 bending cycles, as presented in Figure S19 (Supporting Information). Remarkably, the phosphorescence intensity remains nearly unchanged after cycling, demonstrating excellent mechanical stability, which is crucial for practical applications requiring repeated mechanical deformation. Figure 5b,c shows the phosphorescence spectra and corresponding optical images of the film under varying applied stress levels. A gradual decline in phosphorescence intensity is observed with increasing stress, attributable to the disruption of hydrogen‐bonded interactions between the cyclodextrin and CNDs, which weakens the rigid microenvironment necessary for suppressing non‐radiative decay of triplet states. As shown in Figure S20 (Supporting Information), the phosphorescence intensity exhibits a stress‐dependent characteristic, demonstrating the mechano‐responsive nature of the film and its potential as a stress sensor. Considering the mechano‐responsive characteristics of stress recording film, we envision the film as an optical sensing unit for aircraft wing fatigue monitoring (Figure 5d). In this conceptual application, the film is applied in critical structural components of the aircraft, where it functions as a non‐invasive stress sensor. By leveraging its stress‐sensitive phosphorescence response, the film enables non‐invasive, persistent visualization of stress distribution. Specifically, during flight operations, mechanical stress applied to the aircraft wing structure leads to a localized decrease in phosphorescence intensity, which directly correlates with the magnitude of the applied stress (Figure 5e). These intensity variations can be readily captured and quantified using imaging techniques, enabling real‐time and continuous monitoring of structural fatigue. Specifically, the luminescence image of the CND‐PVA film was converted into a grayscale image (Figure 5f). By employing a specific threshold, the grayscale image can be digitized into quaternary coding (Figure 5g), wherein the bit values assigned to the three pixels were 3, 2, and 1, respectively. For clarity, the partial magnified image consisting of 50 × 50 pixels was extracted for analysis (Figure 5h), and the corresponding quaternary coding maps are shown in Figure 5i. Additionally, a region of 10 × 10 pixels, intercepted from the mark square region, as shown in Figure 5j, shows the complete gray value picture of the image of the CND‐PVA film, displaying gray values across different pixels, which represents the magnitude and distribution of the force. In addition, the film enables persistent and visible detection of low‐velocity impacts, which are often difficult to identify through routine inspections. Specifically, upon a low‐energy impact event, the localized mechanical force induces an immediate and quantifiable decrease in the phosphorescence intensity at the point of impact (Figure 5k). This intensity decrease directly correlates with the impact energy. The resulting phosphorescence distribution pattern can be captured and processed using an imaging system. The luminescence image of the impacted film was first converted into a grayscale image (Figure 5i). By applying a specific threshold, the grayscale image was further digitized into quaternary coding (Figure 5j), where the bit values of 3, 2, and 1 were assigned to represent varying degrees of intensity loss. For detailed analysis, a magnified region of 50 x 50 pixels was extracted (Figure 5n), and its corresponding quaternary code map was generated (Figure 5o). Furthermore, a representative zone of 10 x 10 pixels within the impacted area was analyzed (Figure 5p), displaying the complete gray value matrix. This matrix provides a quantitative digital footprint of the impact, precisely mapping the force distribution and magnitude across the affected region, thereby facilitating rapid impact localization and severity assessment. Over time, regions exhibiting pronounced phosphorescence quenching may serve as early warning indicators of accumulated fatigue or potential mechanical failure. This memory‐capable approach offers a novel method for structural health monitoring, with the potential to significantly improve both safety assurance and maintenance efficiency in aerospace systems. The integration of such mechano‐responsive films onto critical structural components could transform current paradigms for tracking mechanical degradation across a wide range of dynamic and high‐performance environments.

**Figure 5:**
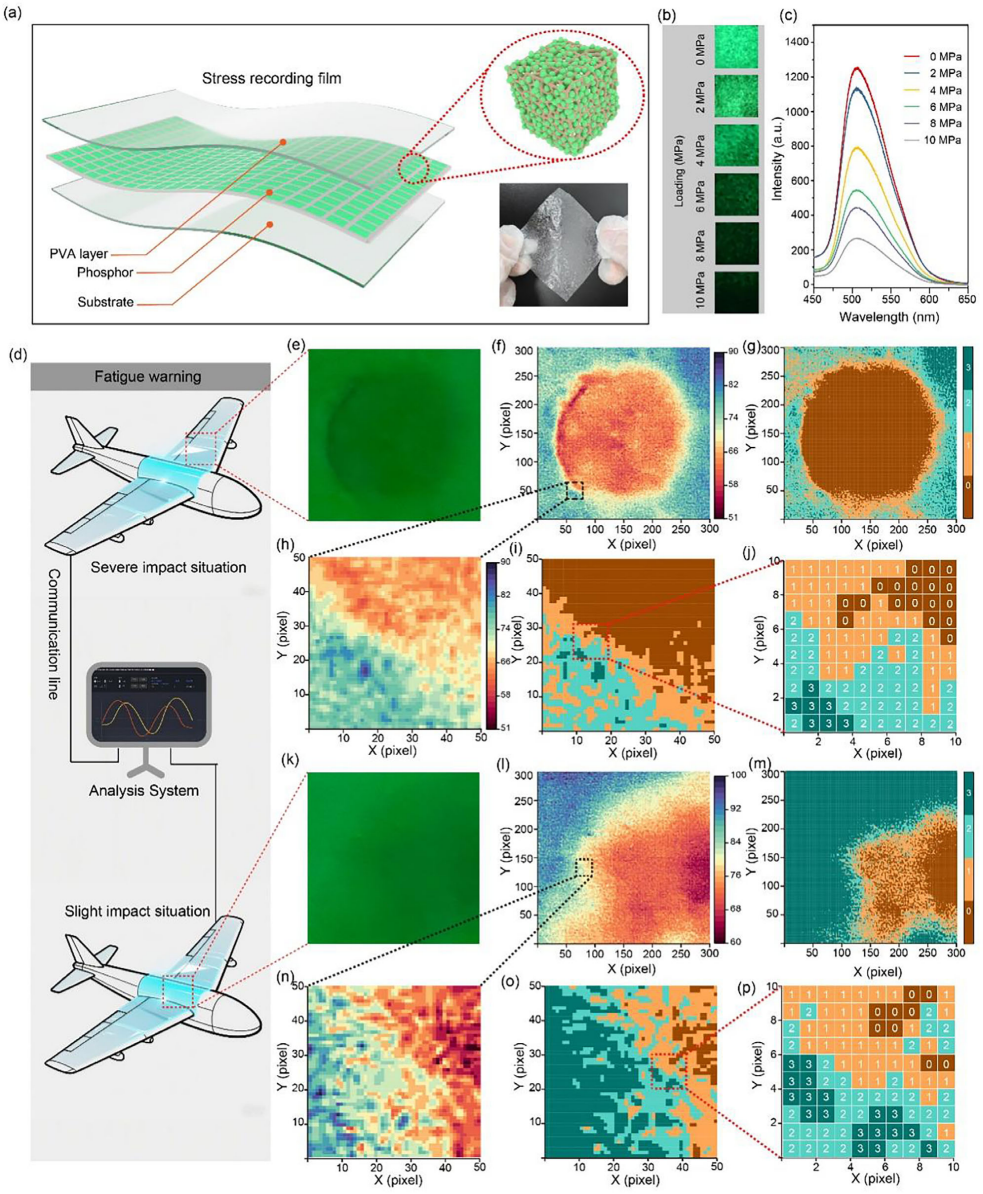
Demonstration of stress‐recording and mechano‐sensing application using a flexible CND‐PVA film. a) Schematic illustration of the fabrication of the CND‐PVA film. b) Top: Photoluminescence spectra of CND‐PVA film; Bottom: Phosphorescence decay profile of the CND‐PVA film. c) Phosphorescence intensity after 500 bending cycles. d) Photographic images of CND‐PVA film after different mechanical stresses. e) Corresponding phosphorescence spectra of CND‐PVA film under varying applied stress levels. f) Plot of phosphorescence intensity versus applied stress. g) The model diagram of the optical sensing unit for aircraft wing fatigue monitoring. h–m) Application of stress distribution of aircraft components based on changes in phosphorescence intensity during operational load.

In summary, we have successfully developed a cyclodextrin‐trapped CNDs system with mechano‐responsive reversible RTP characteristics through hydrogen‐bond‐induced supramolecular assembly. The dynamic confinement framework enables mechano‐responsive phosphorescence quenching via distortion of the hydrogen‐bond network, which enhances non‐radiative decay of triplet excitons under mechanical stimulation. Crucially, the metastable hydrogen‐bond interactions allow recovery of RTP intensity through ultrasonic reconstruction of the rigid cyclodextrin matrix, achieving a reversible “stress‐recording” without covalent bond cleavage. This material may be leveraged for aerospace applications, where the CND‐embedded film demonstrated memorable visualization of stress distribution in wings under sudden mechanical events (e.g., micro‐impacts). This work establishes a non‐destructive structural health monitoring paradigm for extreme aerospace environments, highlighting the potential of hydrogen‐bond‐driven reversible interactions in designing next‐generation smart materials for high‐stress operational sensing.

## Conflict of Interest

The authors declare no conflict of interest.

## Author Contributions

Y.L. conceived the idea; K. L. and Y. L. supervised and coordinated all aspects of the project. H.S., Q. C., L. J. carried out synthesis and characterization, K. L. and Y. L. wrote the paper. H. J., L. K., commented the paper and all authors discussed the results.

## Supporting information



Supporting Information

## Data Availability

Research data are not shared.

## References

[1] K. Jiang , L. Zhang , J. F. Lu , C. X. Xu , C. Z. Cai , H. W. Lin , Angew. Chem. Int. Ed. 2016, 55, 7231. 10.1002/anie.201602445 27135645

[2] S. Kim , F. Yang , H. Jung , G. Hong , S. Hahn , Adv. Funct. Mater. 2024, 34, 2314861.

[3] N. N. Wang , M. J. Pu , Z. D. Ma , Y. G. Feng , Y. F. Guo , W. L. Guo , Y. B. Zheng , L. Q. Zhang , Z. F. Wang , M. Feng , X. J. Li , D. A. Wang , Nano Energy. 2021, 90, 106646.

[4] B. Yoon , T. Oh , Y. Chang , J. Suhr , Small 2024, 23, 2310682. 10.1002/smll.202310682 39109576

[5] Z. J. Song , Y. Shang , Q. Lou , J. Y. Zhu , J. H. Hu , W. Xu , C. C. Li , X. Chen , K. K. Liu , C. X. Shan , X. Bai , Adv. Mater. 2023, 35, 2207970. 10.1002/adma.202207970 36413559

[6] S. Yagai , S. Okamura , Y. Nakano , M. Yamauchi , K. Kishikawa , T. Karatsu , A. Kitamura , A. Ueno , D. Kuzuhara , H. Yamada , T. Seki , H. Ito , Nat. Commun. 2014, 5, 4013. 24898172 10.1038/ncomms5013

[7] Y. C. Liang , H. C. Shao , K. K. Liu , Q. Cao , S. F. Zhang , H. Y. Wang , L. Y. Jiang , C. X. Shan , L. M. Kuang , H. Jing , ‌Light Sci. Appl. 2025, 14, 316. 40935832 10.1038/s41377-025-01965-0 PMC12426232

[8] Y. C. Liang , Q. Cao , Y. Deng , Y. Wang , K. K. Liu , C. X. Shan , Nano Res. 2024, 17, 6534.

[9] Y. C. Liang , H. C. Shao , K. K. Liu , Q. Cao , L. Y. Jiang , C. X. Shan , L. M. Kuang , H. Jing , Small 2024, 20, 2312218. 10.1002/smll.202312218 38716754

[10] T. Mutai , S. Takamizawa , J. Photoch. Photobio. C. 2022, 51, 100479.

[11] Q. Lou , X. G. Yang , K. K. Liu , Z. Z. Ding , J. X. Qin , Y. Z. Li , C. F. Lv , Y. Shang , Y. W. Zhang , Z. F. Zhang , J. H. Zang , L. Dong , C. X. Shan , Nano Res. 2022, 15, 2545.

[12] J. Z. Chen , F. X. Lin , D. M. Guo , T. Y. Tang , Y. L. Miao , Y. W. Wu , W. T. Zhai , H. H. Huang , Z. G. Chi , Y. M. Chen , Z. Y. Yang , Adv. Mater. 2024, 36, 2409642. 10.1002/adma.202409642 39466913

[13] S. Y. Song , K. K. Liu , X. Mao , Q. Cao , N. Li , W. B. Zhao , Y. Wang , Y. C. Liang , J. H. Zang , X. Li , Q. Lou , L. Dong , C. X. Shan , Adv. Mater. 2023, 35, 2212286. 10.1002/adma.202212286 36840606

[14] Y. C. Liang , K. K. Liu , X. Y. Wu , Q. Lou , L. Z. Sui , L. Dong , K. J. Yuan , C. X. Shan , Adv. Sci. 2021, 8, 2003433. 10.1002/advs.202003433 PMC7967062 33747738

[15] S. Y. Song , K. K. Liu , Q. Cao , X. Mao , W. B. Zhao , Y. Wang , Y. C. Liang , J. H. Zang , Q. Lou , L. Dong , C. X. Shan , Light Sci. Appl. 2022, 11, 146. 35595762 10.1038/s41377-022-00837-1 PMC9122994

[16] F. Nie , D. Yan , Nat. Commun. 2024, 15, 9491. 39488522 10.1038/s41467-024-53963-2 PMC11531476

[17] G. Pacchioni , Nat. Rev. Mater. 2023, 8, 362.

[18] Z. Z. Li , S. Cao , Y. Y. Zheng , L. Q. Song , H. C. Zhang , Y. L. Zhao , Adv. Funct. Mater. 2024, 34, 2306956.

[19] X. Dai , M. Huo , Y. Liu , Nat. Rev. Chem. 2023, 7, 854. 37993737 10.1038/s41570-023-00555-1

[20] X. L. Zhou , X. Bai , F. J. Shang , H. Y. Zhang , L. H. Wang , X. F. Xu , Y. Liu , Nat. Commun. 2024, 15, 4787. 38839843 10.1038/s41467-024-49238-5 PMC11153566

[21] X. K. Ma , W. Zhang , Z. X. Liu , H. Y. Zhang , B. Zhang , Y. Liu , Adv. Mater. 2021, 33, 2007476.

[22] X. Ma , Y. Liu , Acc. Chem. Res. 2021, 54, 3403. 34403251 10.1021/acs.accounts.1c00336

[23] W. L. Zhou , Y. Chen , Q. L. Yu , H. Y. Zhang , Z. X. Liu , X. Y. Dai , J. J. Li , Y. Liu , Nat. Commun. 2020, 11, 4655. 32938918 10.1038/s41467-020-18520-7 PMC7494876

[24] Y. C. Liang , H. C. Shao , K. K. Liu , Q. Cao , Y. Deng , Y. W. Hu , K. Yang , L. Y. Jiang , C. X. Shan , L. M. Kuang , H. Jing , ACS Appl. Mater. Interfaces. 2024, 16, 26643. 38716902 10.1021/acsami.4c00081

[25] L. Y. Jiang , Y. C. Zhou , S. F. Zhang , H. C. Shao , Y. C. Liang , Nano Lett. 2024, 24, 8418. 38934472 10.1021/acs.nanolett.4c02165

[26] Z. Yin , Z. Wu , B. Liu , Adv. Mater. 2025, 37, 2506549. 10.1002/adma.202506549 40465363

[27] W. S. Xu , G. Y. Bai , T. T. Li , L. Gao , X. L. Yan , Y. Li , L. G. Chen , B. W. Wang , Nat. Commun. 2025, 16, 4189. 40328754 10.1038/s41467-025-59360-7 PMC12056082

[28] L. H. Hou , T. Wang , S. F. Yu , X. H. Xu , X. Yu , Aggregate 2025, 6, 70071.

[29] H. Chen , Y. Y. Zhang , J. Y. Shan , M. Y. Dong , Z. Qian , A. Q. Lv , H. J. Qian , H. L. Ma , Z. F. An , L. Gu , W. Huan , Angew. Chem., Int. Ed. 2025, 64, 202500610. 10.1002/anie.202500610 39933998

[30] C. S. Li , Z. C. Lou , M. H. Wu , F. L. Ma , X. M. Chen , B. Z. Tang , J. Am. Chem. Soc. 2025, 147, 18317. 40377380 10.1021/jacs.5c06288

[31] X. Han , H. Q. Zheng , Y. Yang , Y. Cui , G. Qian , Adv. Funct. Mater. 2025, 35, 2425934.

[32] X. Y. He , W. L. Huang , Y. H. Zheng , X. K. Xu , H. P. Wei , P. Liang , X. F. Yang , C. F. Hu , X. J. Zhang , B. F. Lei , X. C. Zhang , J. T. Ye , Y. L. Liu , J. L. Zhuang , Angew. Chem., Int. Ed. 2025, 64, 202423388. 10.1002/anie.202423388 39907178

[33] W. Zhao , Z. He , B. Z. Tang , Nat. Rev. Mater. 2020, 5, 869.

[34] W. P. Ye , H. L. Ma , H. F. Shi , H. Wang , A. Q. Lv , L. F. Bian , M. Zhang , C. Q. Ma , K. Ling , M. X. Gu , Y. F. Mao , X. K. Yao , C. F. Gao , K. Shen , W. Y. Jia , J. H. Zhi , S. Z. Cai , Z. C. Song , J. J. Li , Y. Y. Zhang , S. Lu , K. Liu , C. M. Dong , Q. Wang , W. Huang , Nat. Mater. 2021, 20, 1539. 34426660 10.1038/s41563-021-01073-5

[35] Y. C. Liang , Y. Shang , K. K. Liu , Z. Liu , W. J. Wu , Q. Liu , Q. Zhao , X. Y. Wu , L. Dong , C. X. Shan , Nano Res. 2020, 13, 875.

[36] X. L. Zhou , X. Zhao , X. Bai , Q. W. Cheng , Y. Liu , Adv. Funct. Mater. 2024, 34, 2400898.

[37] S. József , Chem. Rev. 2010, 29, 1743.

[38] N. Ganesh , J. L. Shen , M. Ishita , S. Abhay , B. Ramiz , Prog. Mater. Sci. 2022, 124, 100869.

[39] M. E. Davis , M. E. Brewster , Nat. Rev. Drug Discovery 2004, 3, 1023. 15573101 10.1038/nrd1576

[40] G. Crini , Chem. Rev. Review: A History of Cyclodextrins. 2014, 114, 10940. 10.1021/cr500081p 25247843

[41] K. A. Connors , Chem. Rev. 1997, 97, 1325. 11851454 10.1021/cr960371r

[42] Q. Gao , B. Z. Lv , F. Peng , Prog. Mater. Sci. 2025, 148, 101372.

[43] D. Li , Y. j. Yang , J. Yang , M. M. Fang , B. Z. Tang , Z. Li , Nat. Commun. 2022, 13, 347. 35039504 10.1038/s41467-022-28011-6 PMC8764117

[44] M. Richard‐Lacroix , V. Deckert , Light Sci. Appl. 2020, 9, 35. 32194949 10.1038/s41377-020-0260-9 PMC7061098

[45] Y. C. Liang , S. S. Gou , K. K. Liu , W. J. Wu , C. Z. Guo , S. Y. Lu , J. H. Zang , X. Y. Wu , Q. Lou , L. Dong , Y. F. Gao , C. X. Shan , Nano Today 2020, 34, 100900.

[46] D. J. Xu , M. L. Wang , R. Z. Huang , J. F. Stoddart , Y. P. Wang , J. Am. Chem. Soc. 2025, 147, 4450. 39849299 10.1021/jacs.4c15655

[47] S. Chakraborty , S. Choudhury , N. K. Singha , Small 2024, 20, 2406866. 39258360 10.1002/smll.202406866 PMC11600686

[48] Z. J. Qiu , W. J. Zhao , M. K. Cao , Y. Q. Wang , J. W. Y. Lam , Z. Zhang , X. Chen , B. Z. Tang , Adv. Mater. 2018, 30, 1803924. 10.1002/adma.201803924 30260534

[49] Q. Liu , S. S. Yue , Z. Q. Yan , Y. F. Xie , H. Cai , Chin. J. Struct. Chem. 2022, 41, 2204075.

[50] D. Yang , Y. Y. Ren , J. W. Li , Q. Wang , X. B. Li , X. Z. Qu , Chem. Eng. J. 2022, 431, 133449.

[51] F. Nie , B. Zhou , D. P. Yan , Chem. Eng. J. 2023, 453, 139806.

