# Supplementary material for: Reversible Stress‐memory Phosphorescent Carbon Nanodots via Supramolecular Confinement Engineering for Aerospace Monitoring
Source: Adv Sci (Weinh). 2025 Dec 12;13(12):e21219. doi: 10.1002/advs.202521219 (PMC12948269; doi:10.1002/advs.202521219)
Supplement: Supplementary file 1 — Supporting Information [file ADVS-13-e21219-s001.doc]

Supplementary Information

**Reversible Stress-memory Phosphorescent Carbon Nanodots via Supramolecular Confinement Engineering for Aerospace Monitoring**

*Yachuan Liang^1,2,3^, Haochun Shao^1^, Kaikai Liu^4*^, Qing Cao^4^, Liying Jiang^1,2,3*^, Chongxin Shan^4^, Leman Kuang^2,5^, Hui Jing^2,5*^*

^1^ School of Electronics and Information, Zhengzhou University of Light Industry, Zhengzhou 450002, China.

^2^ Academy for Quantum Science and Technology, Zhengzhou University of Light Industry, Zhengzhou 450002, China

^3^ Henan Key Laboratory of Information Functional Materials and Sensing Technology, Zhengzhou University of Light Industry, Zhengzhou 450002, China.

^4^ Henan Key Laboratory of Diamond Optoelectronic Material and Devices, School of Physics and Laboratory of Zhongyuan Light, Zhengzhou University, Zhengzhou 450001, China

^5^ Key Laboratory of Low-Dimensional Quantum Structures and Quantum Control of Ministry of Education, Department of Physics and Synergetic Innovation Center for Quantum Effects and Applications, Hunan Normal University, Changsha 410081, China.

**Materials and methods**

**Materials**

Ethylenediamine (EDA), (purity > 99 %), phosphoric acid (purity > 95 %), Cyclodextrin (purity > 95%) and deionized water. All the chemicals were purchased from Macklin Chemistry Co. Ltd (Shanghai, China). Note that all the chemicals used in this work were analytical grade without further purification.

**Synthesis of CNDs solution**

Firstly, 1.0 mL of EDA solution was dissolved in 15 mL deionized water, and then 2 mL of phosphoric acid was added into the EDA aqueous solution with stirring for 5 min. The formed transparent solution was then heated in a microwave oven (750 W) for 120 s. And then 20 mL deionized water was added into above sample when the sample cooled down to room temperature, and the light yellow solution was obtained. The aqueous solution was centrifuged for 10 min in order to remove sediment. The supernatant was filtered through 0.22 μm membrane and the supernatant was collected.

**Synthesis of cyclodextrin-trapped CNDs**

Disperse 1.4 ml of CNDs into 15 ml of deionized water, add 1.0 g of cyclodextrin, stir for 10 minutes, and then put it into an ultrasonic generator. Under 30 min ultrasonic durations, the cyclodextrin-trapped CNDs can be obtained.

**Quantum yield measurement:**

The quantum yield (QY) of the sample was carried out using an FLS1000 spectrometer equipped with a calibrated integrating sphere. The QY was calculated using the following formula:


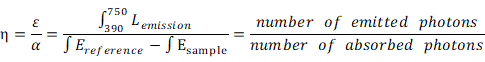


In this equation, *η* represents the quantum yield (QY), *ε* denotes the number of emitted photons from the sample that are captured by the integrating sphere over the 390-750 nm wavelength range, and α signifies the number of photons absorbed by the sample. L_emission_ indicates the net phosphorescent light emitted by the sample. E_reference_ corresponds to the absorption spectrum of the reference material inside the sphere, while E_sample_ refers to the combined absorption spectrum of both the sample and the reference. Based on this equation, the QY of the sample can be calculated.

**Characterization**

The fluorescence and phosphorescence spectra were collected using the Hitachi F-7100 spectrophotometer and FLS1000 spectrometer. TEM images were recorded on JSM-6700F transmission electron microscope. The lifetimes of the CNDs were measured by time-correlated single photon counting on a FLS1000 spectrometer with excitation of 350 nm at room temperature. X-ray diffraction (XRD) patterns were obtained on X’Pert Pro diffractometer.

**
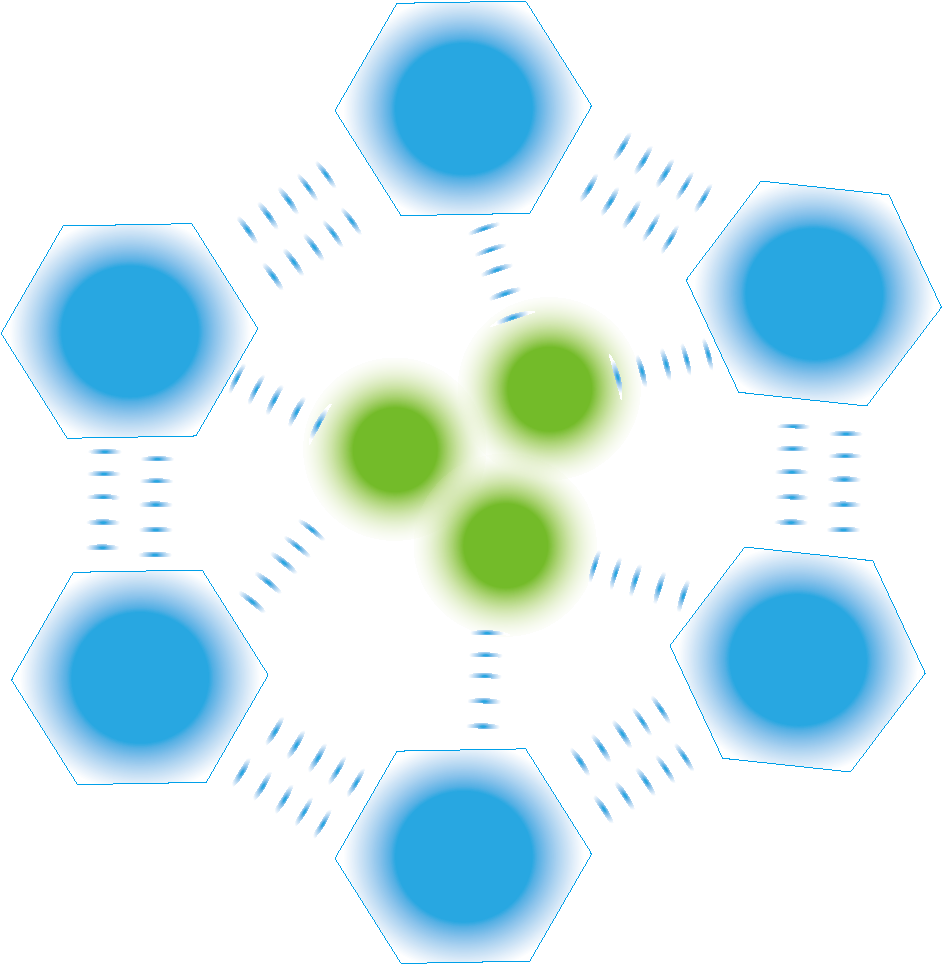
**

**Figure S1** Carbon nanodots (CNDs) in cyclodextrin crystalline frameworks.


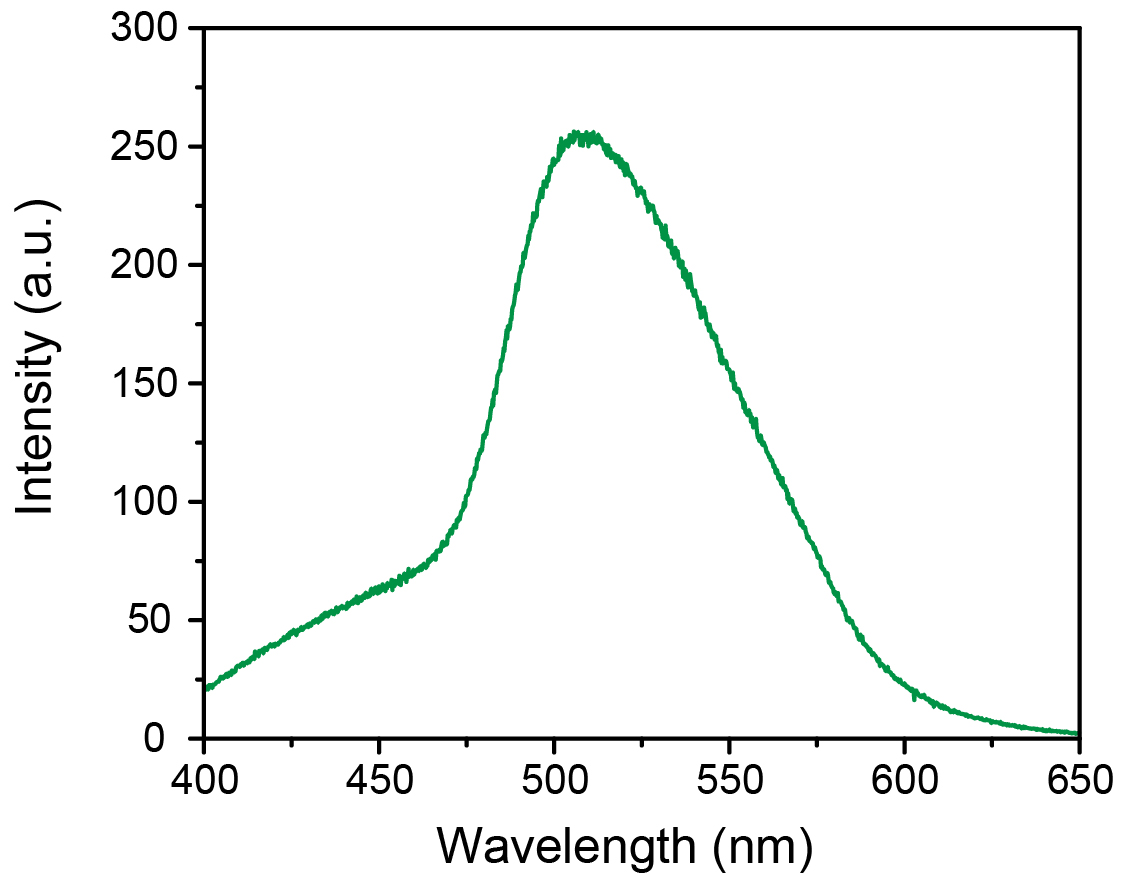


**Figure S2** Phosphorescent spectrum of the cyclodextrin-trapped CNDs.


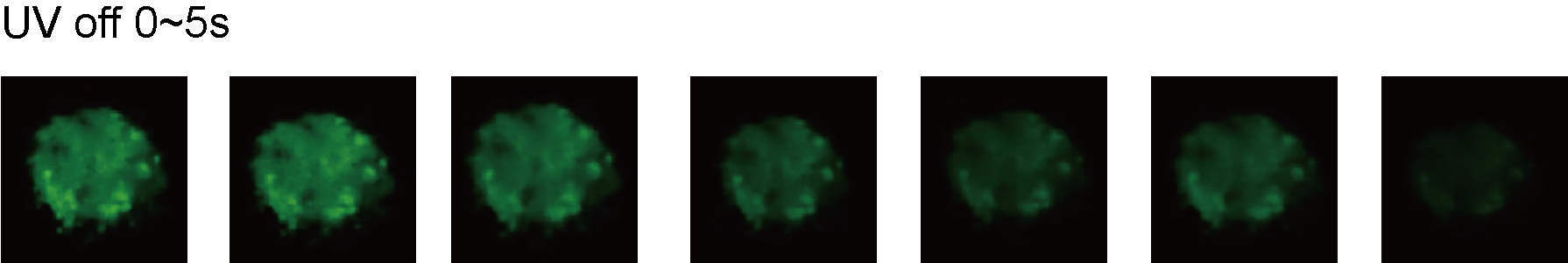


**Figure S3** Phosphorescent photographs of the cyclodextrin-trapped CNDs varying over time.


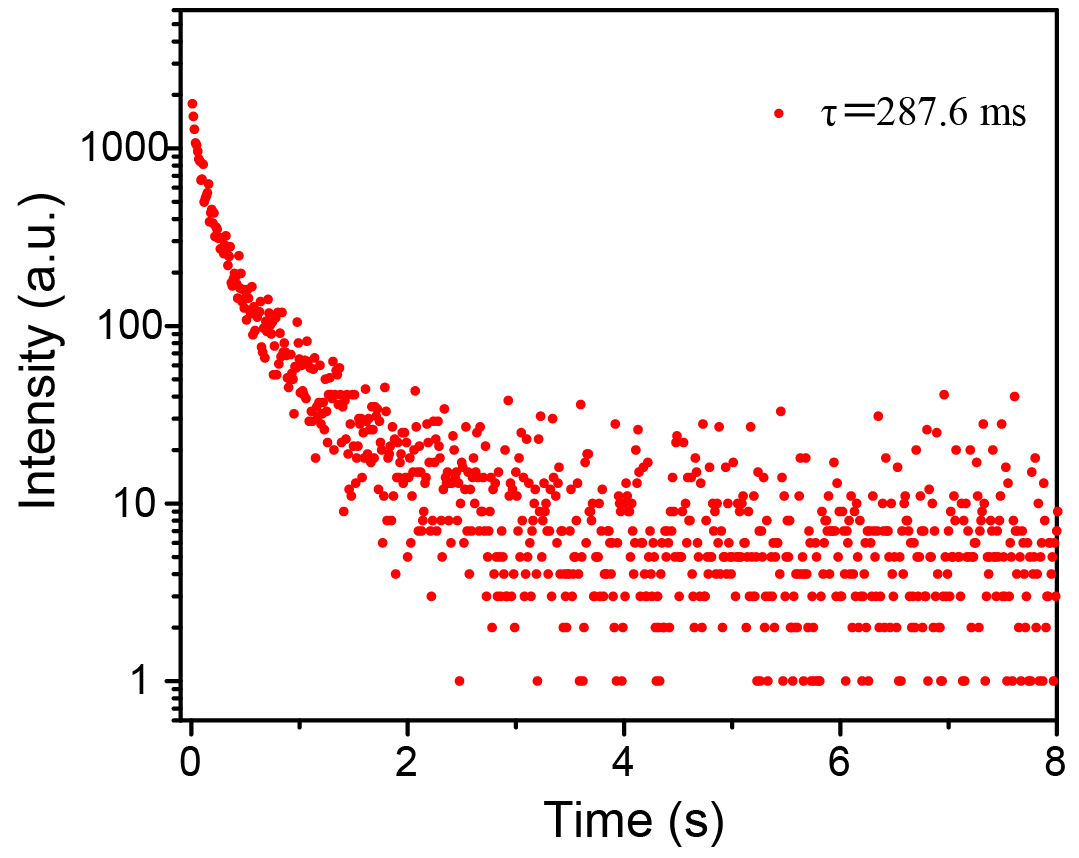


**Figure S4** Phosphorescence Lifetime of the cyclodextrin-trapped CNDs.


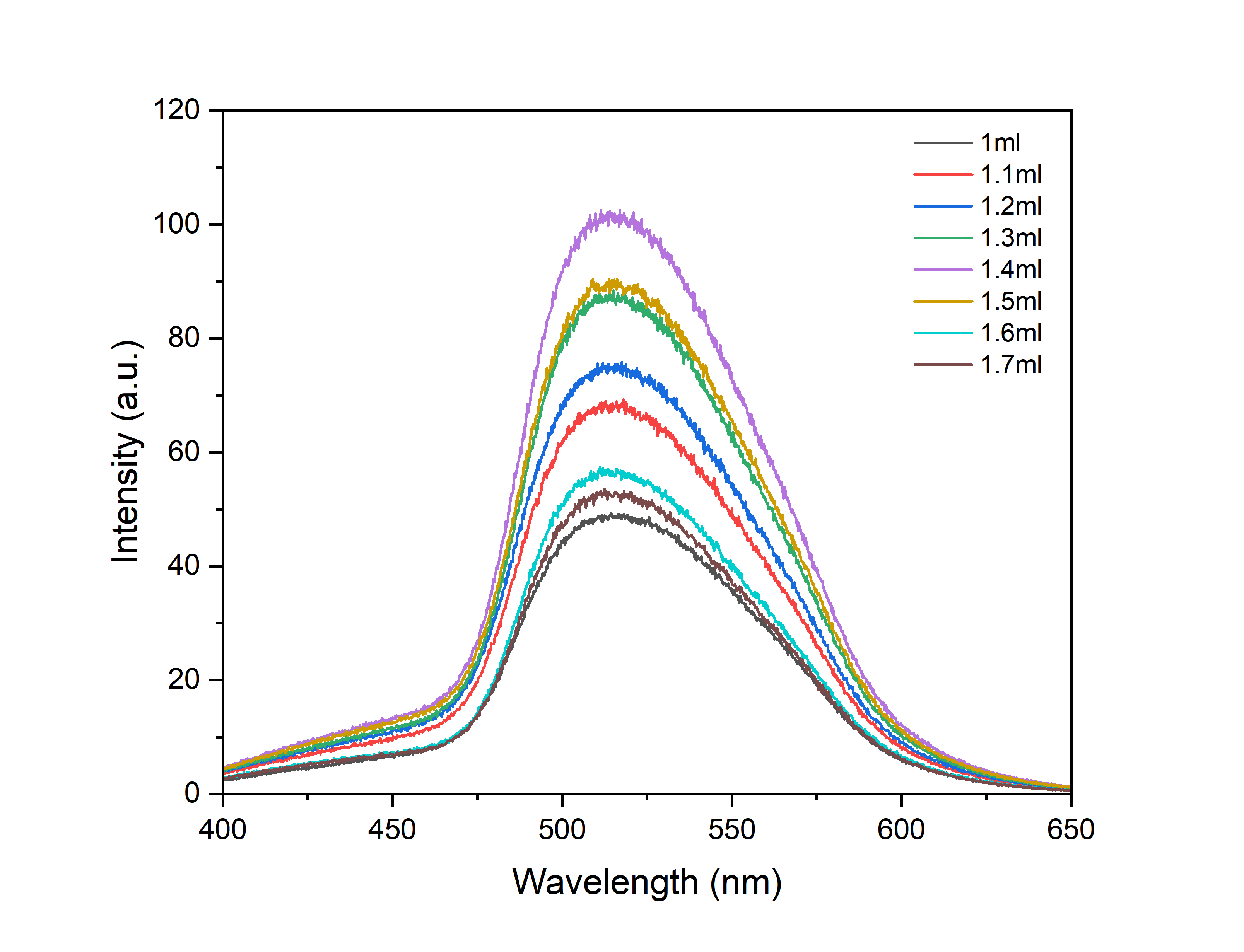


**Figure S5** The phosphorescence intensity of the cyclodextrin-trapped different mass of CNDs.


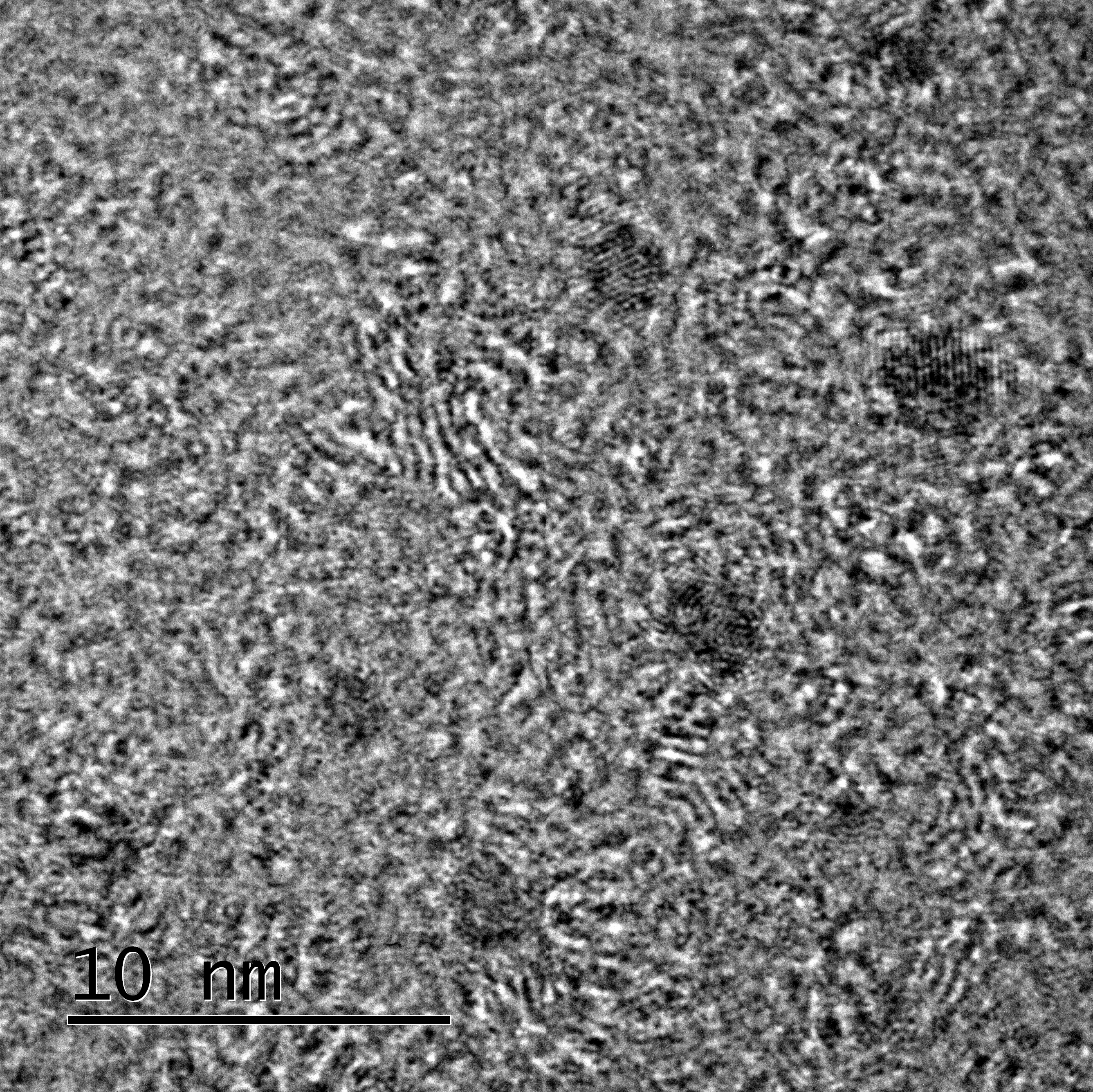


**Figure S6** Transmission electron microscopy of the bare CNDs.


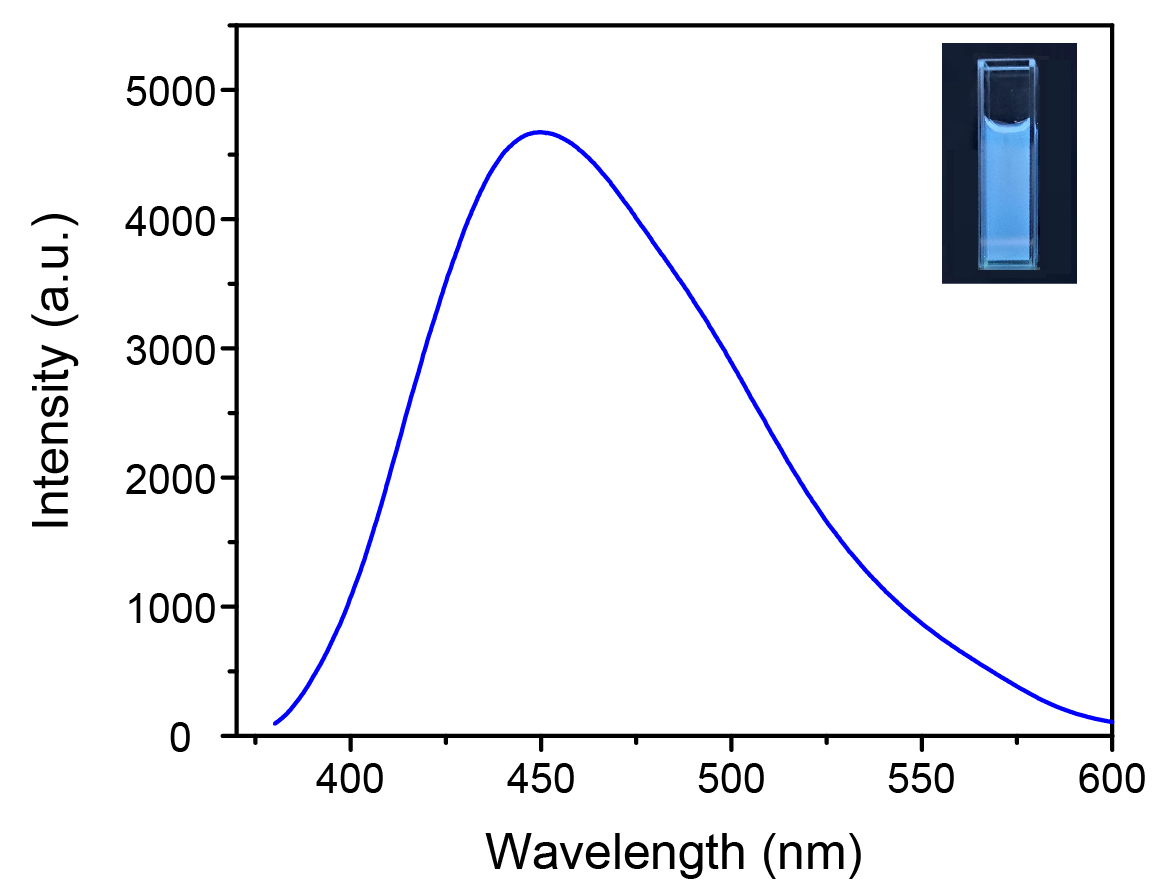


**Figure S7** Fluorescence spectrum of the bare CNDs.


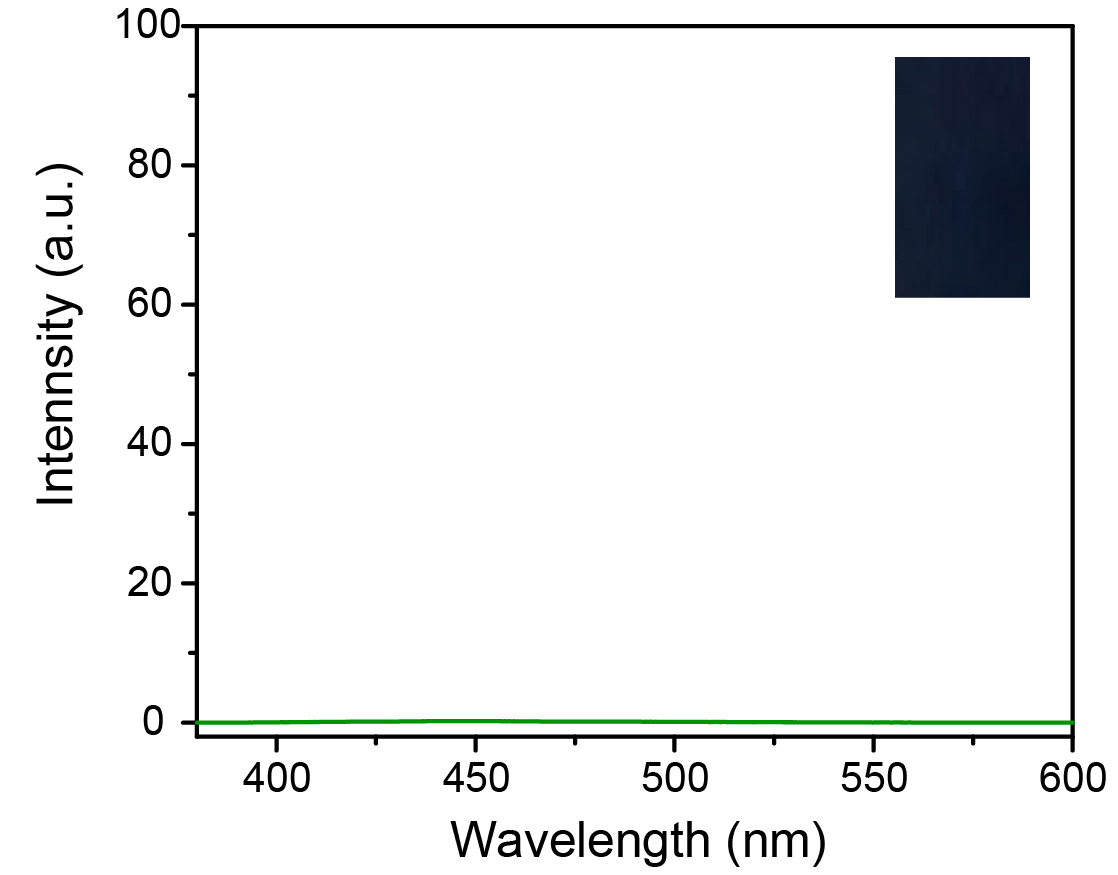


**Figure S8** Phosphorescence spectrum of the bare CNDs.


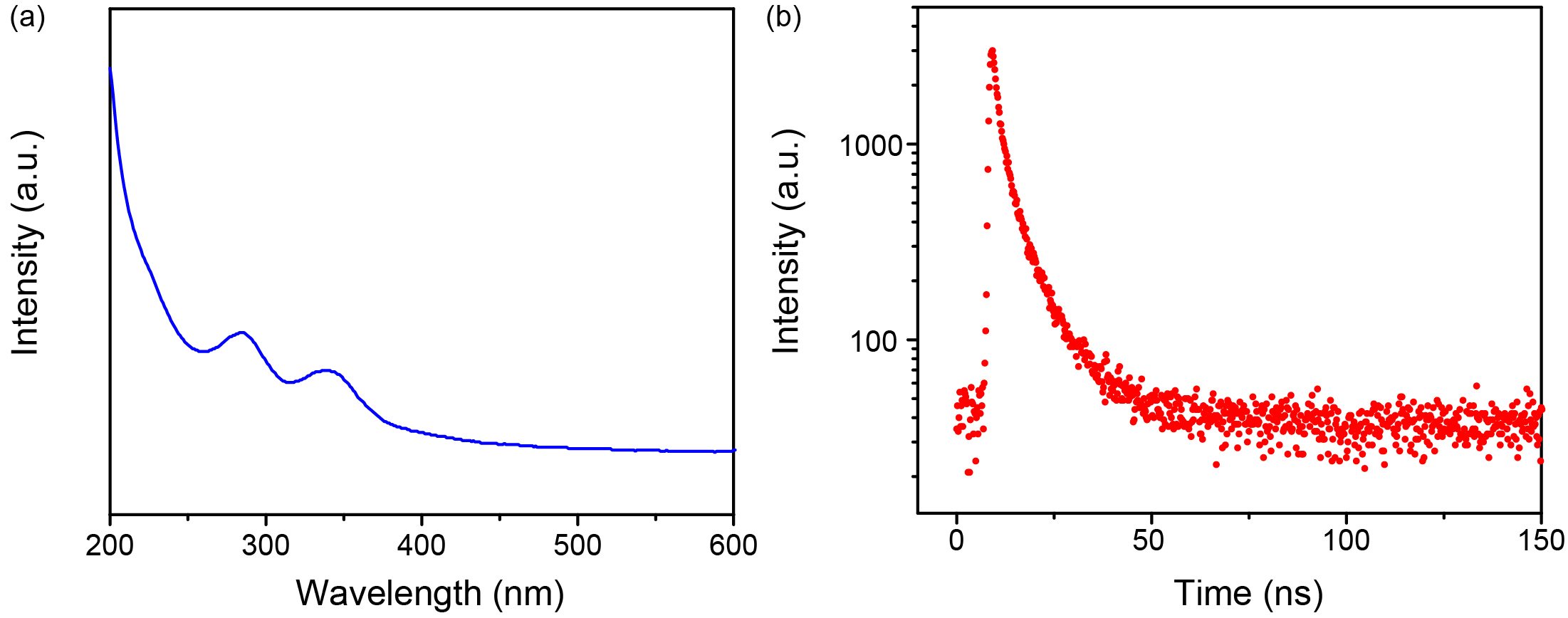


**Figure S9** (a) The UV-vis absorption spectrum of the bare CNDs. (b) Fluorescence lifetime of the bare CNDs.


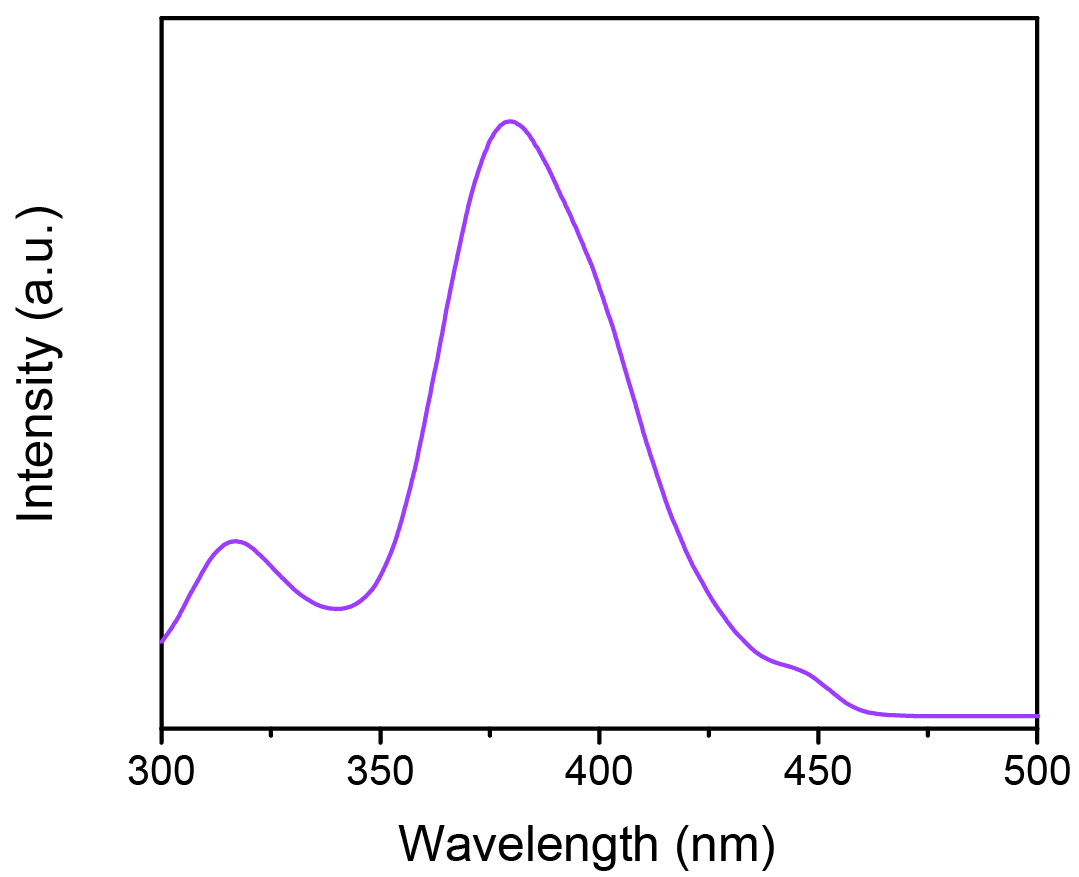


**Figure S10** The excitation spectrum of the bare CNDs.


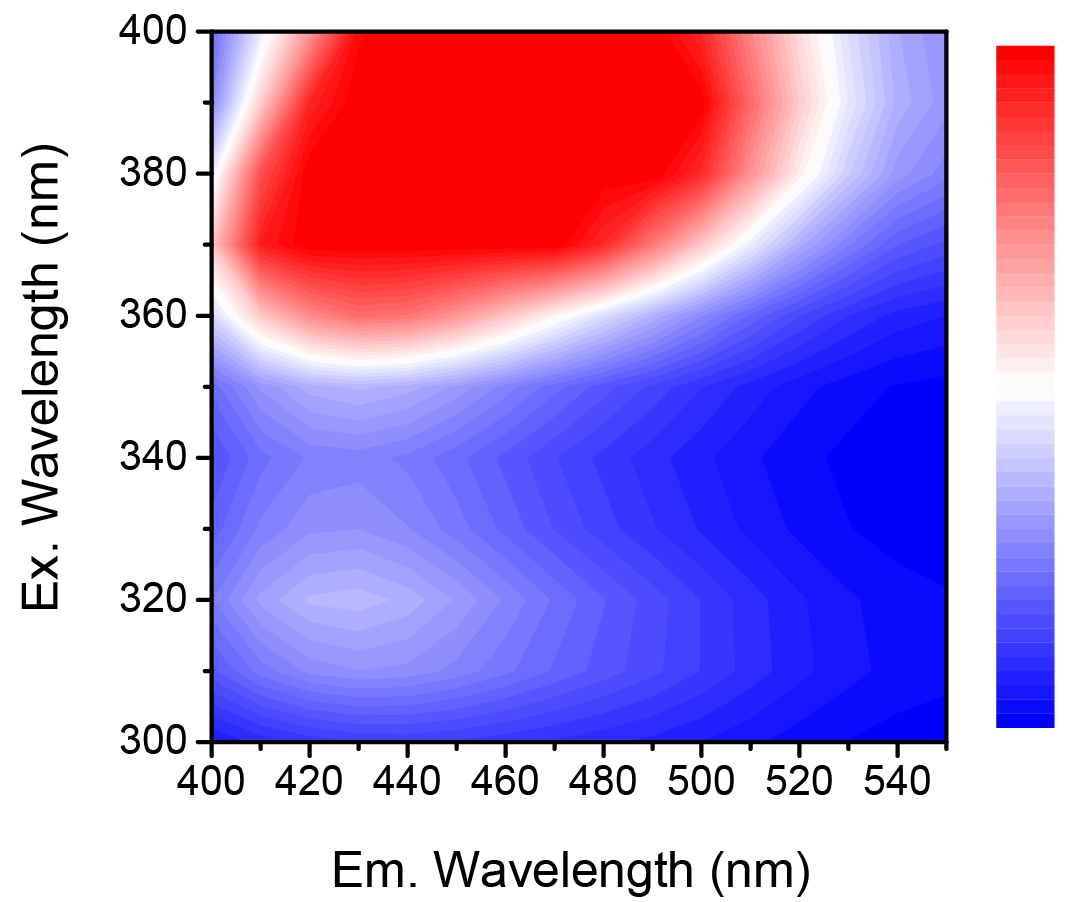


**Figure S11** The excitation-emission contour plot of the bare CNDs.


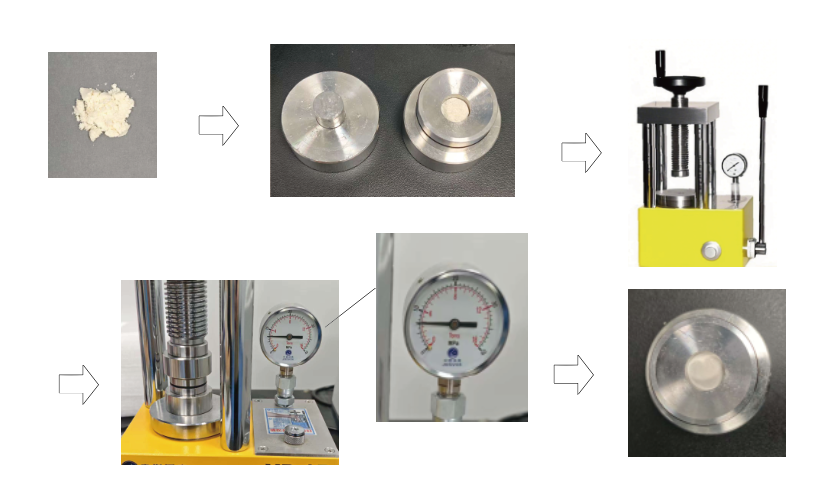


**Figure S12** The custom-designed mechanical compression device.


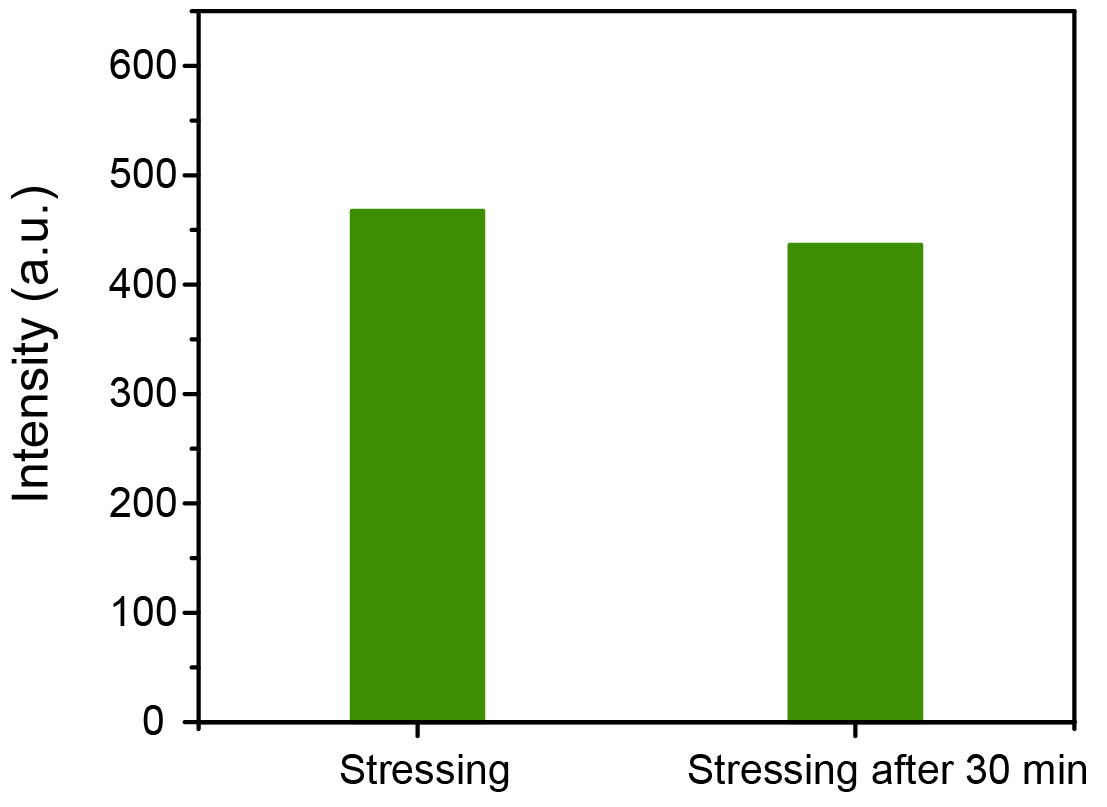


**Figure S13** The spectra of applying stress on CNDs and after 30 minutes of stressing.


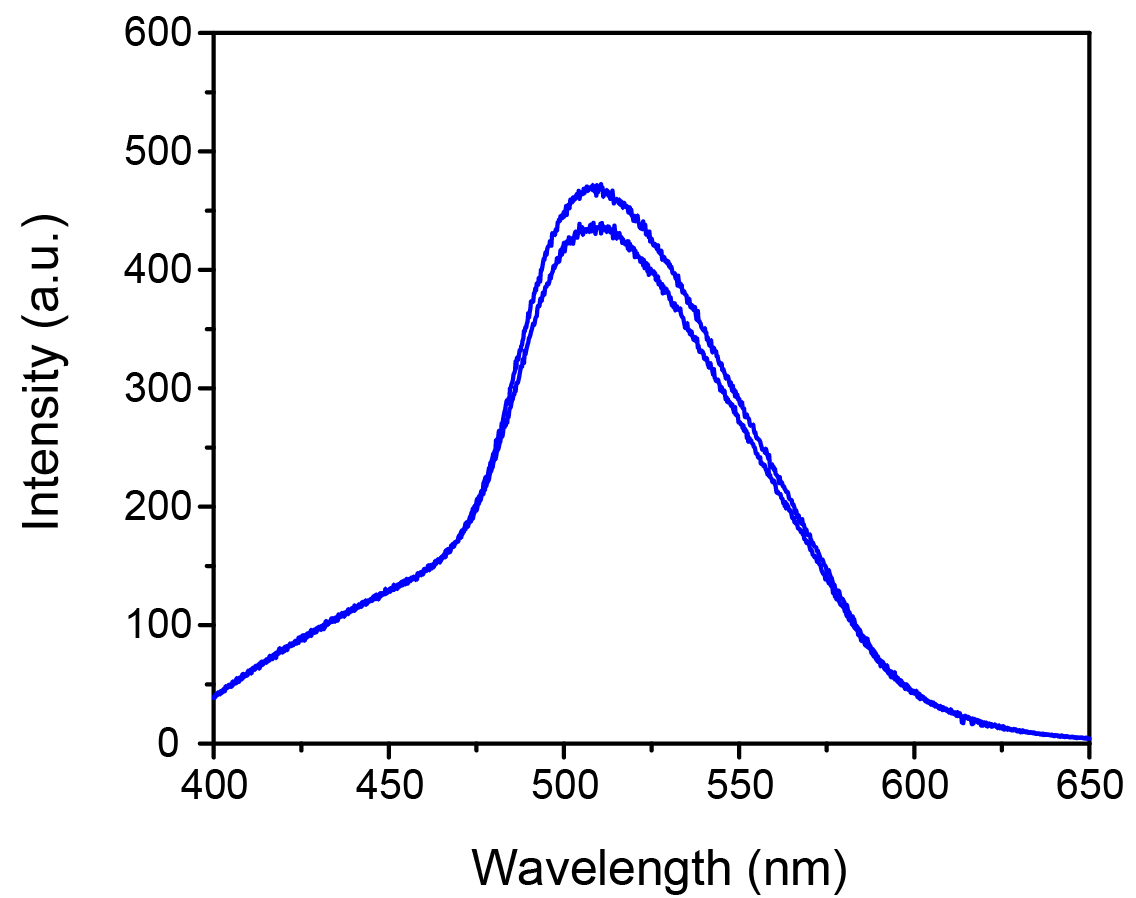


**Figure S14** The emission profile of CNDs before and after stressing.


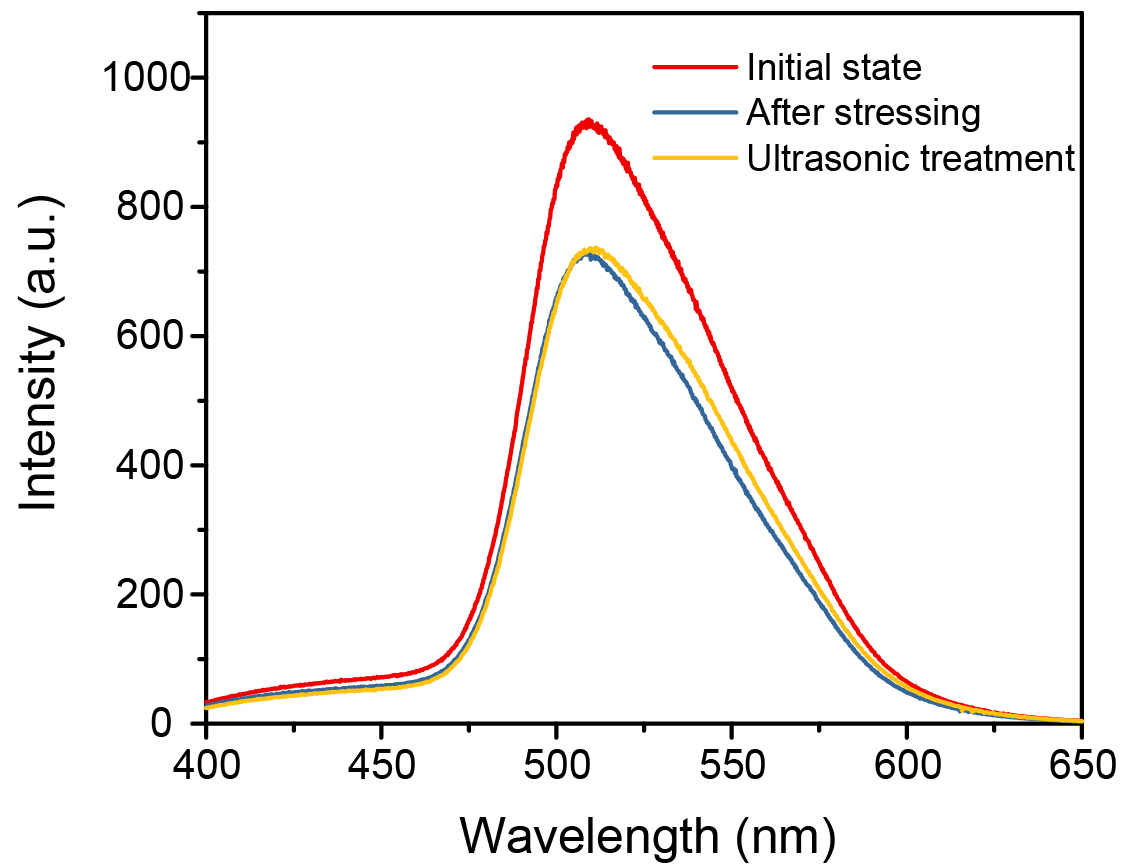


**Figure S15** The spectra of CNDs@SiO₂ powder before and after stressing/ultrasound.


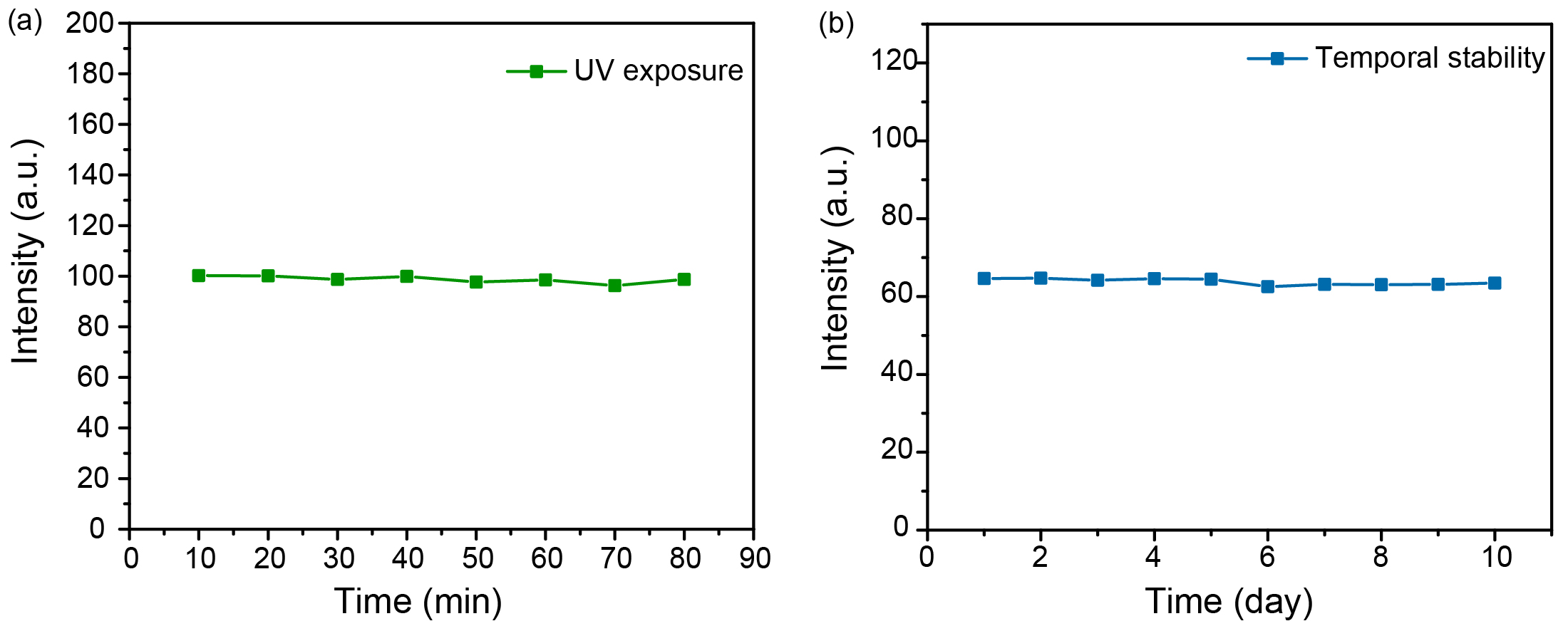


**Figure S16** (a) Changes in phosphorescence intensity of the cyclodextrin-trapped CNDs over time after 80 minutes of UV light irradiation. (b) The phosphorescence intensity of the cyclodextrin-trapped CNDs over days in air.


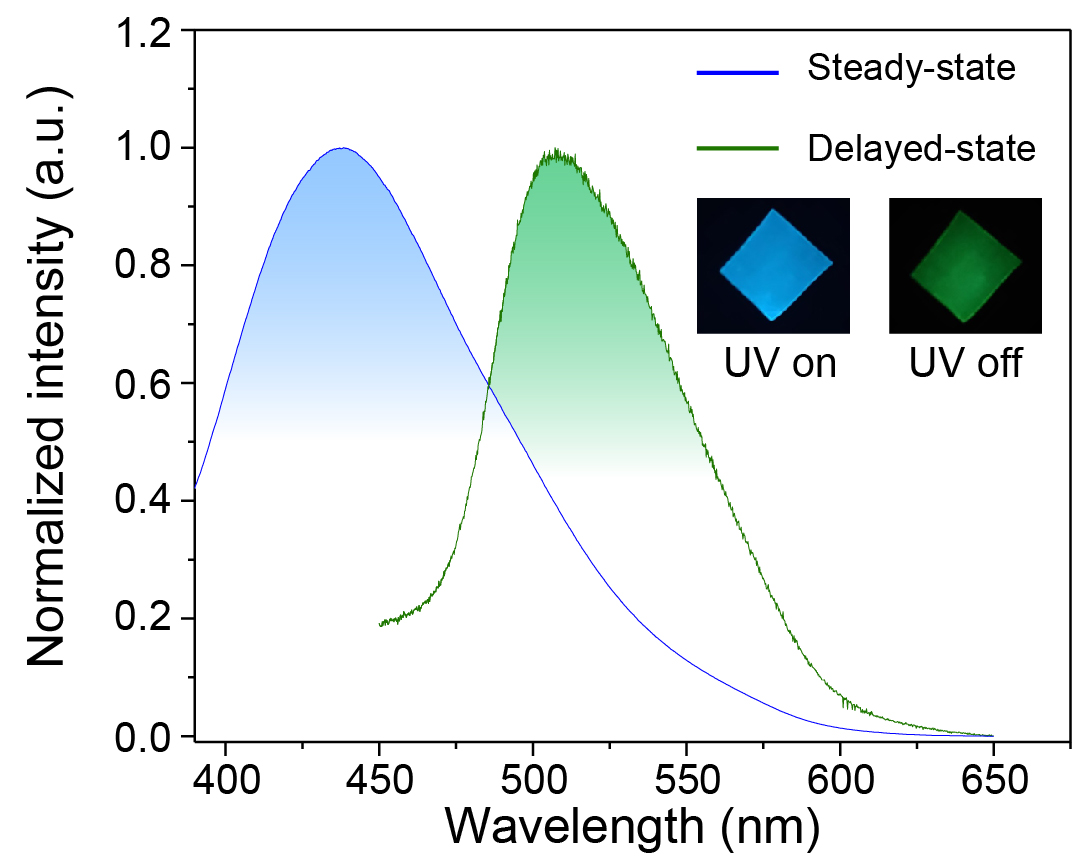


**Figure S17** Photoluminescence spectra of CND-PVA film.


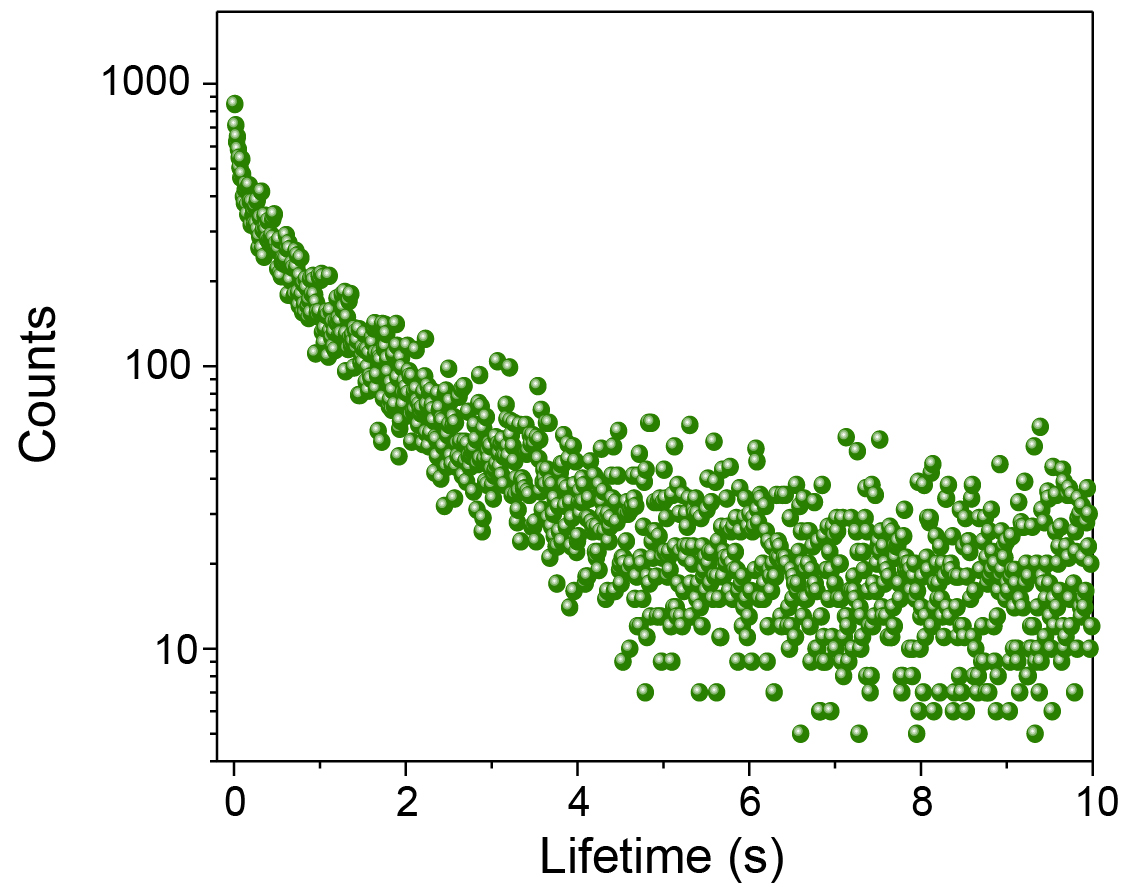


**Figure S18** Phosphorescence decay profile of the CND-PVA film.


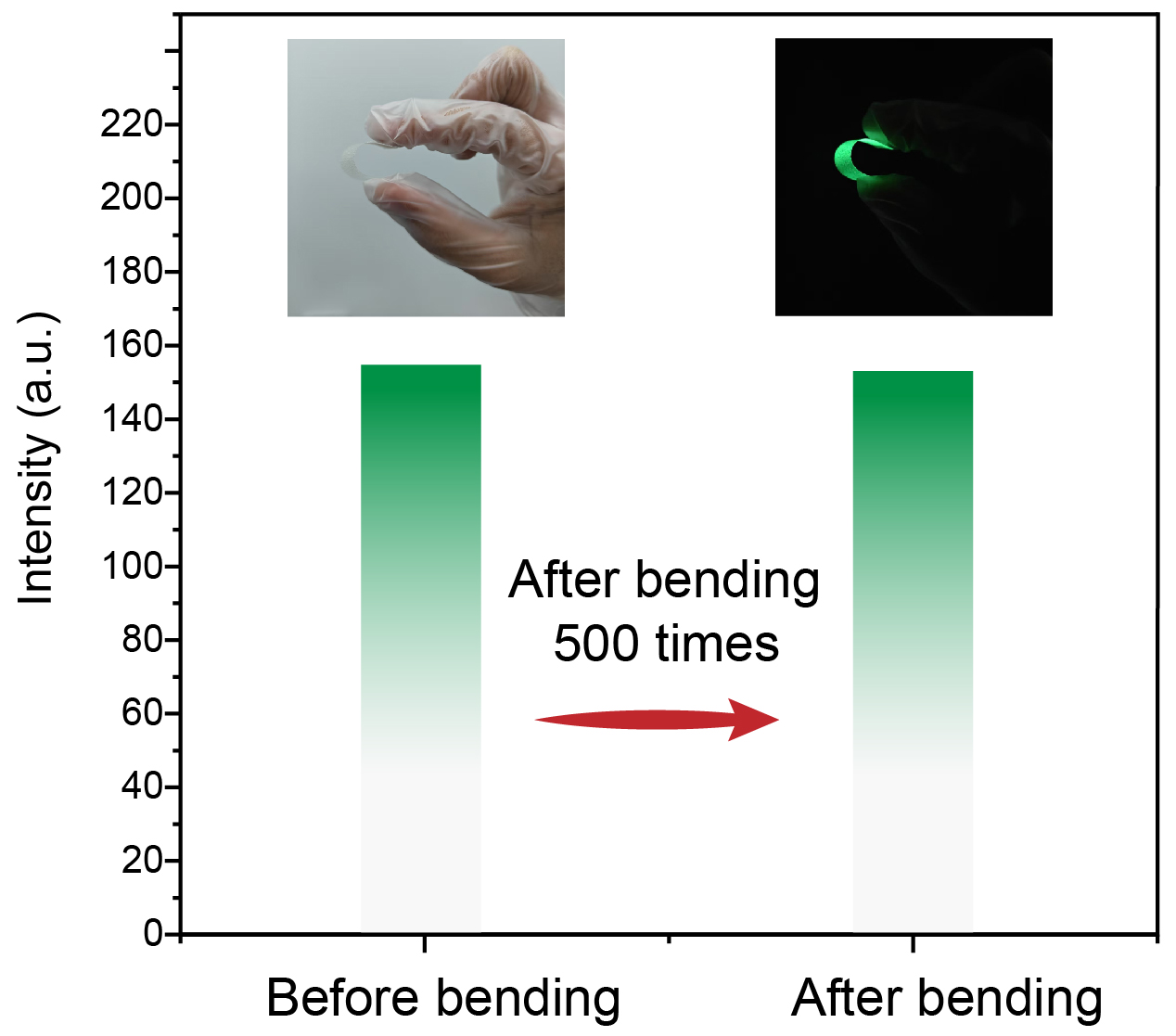


**Figure S19** Phosphorescence intensity after 500 bending cycles.


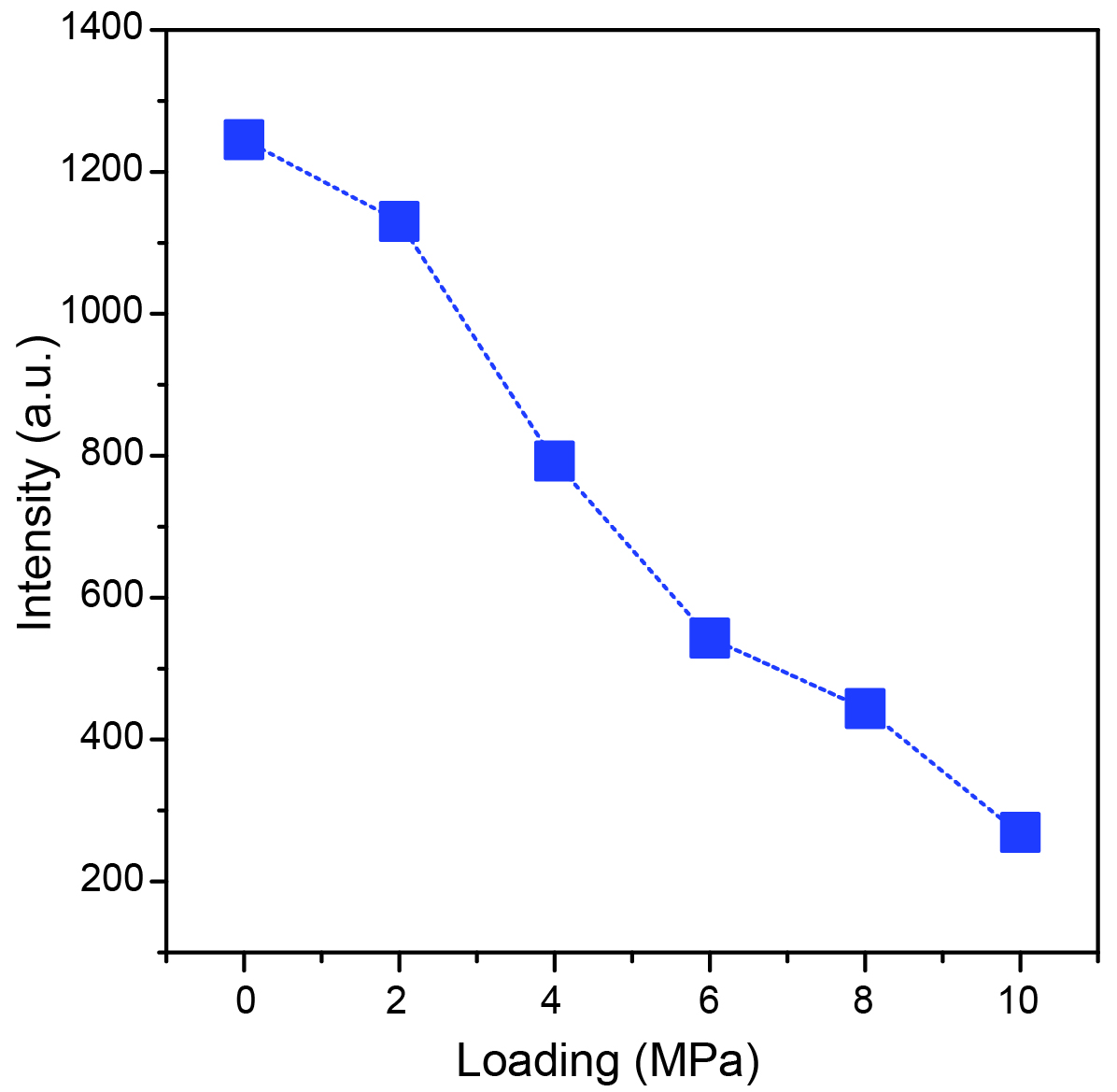


**Figure S20** Plot of phosphorescence intensity versus applied stress.
